# Proteomic analysis of outer membrane vesicles derived from the type A5 Strain of *Mannheimia haemolytica*

**DOI:** 10.3389/fcimb.2025.1578027

**Published:** 2025-06-11

**Authors:** Ke Shang, Yuanji Gao, Jiangbo Du, Chang Liu, Jinglei Dai, Junfeng Zhang, Yanyan Jia, Zuhua Yu, Songbiao Chen, Zhongyu Liu

**Affiliations:** ^1^ The 989th Hospital of the Joint Logistics Support Force of Chinese People’s Liberation Army, Luoyang, China; ^2^ Laboratory of Functional Microbiology and Animal Health, College of Animal Science and Technology, Henan University of Science and Technology, Luoyang, China; ^3^ Luoyang Key Laboratory of Live Carrier Biomaterial and Animal Disease Prevention and Control, Henan University of Science and Technology, Luoyang, China; ^4^ College of Medical Technology and Engineering, Henan University of Science and Technology, Luoyang, China

**Keywords:** *Mannheimia haemolytica* type A5, outer membrane vesicles, proteomics, inflammatory response, *in vitro* test

## Abstract

*Mannheimia haemolytica* (*M. haemolyt*ica) cause mastitis in sheep, acute sepsis in newborn lambs, and co-infections with various pathogens, leading to bovine respiratory disease syndrome (BRDS), these infections have resulted in significant economic losses to both domestic and international farming industries. An in-depth understanding of the pathogenic mechanisms of *M. haemolytica* is crucial for the prevention and control of this disease. Outer membrane vesicles (OMVs) play a vital role in bacterial pathogenesis, serving as key mediators of interactions between Gram-negative bacteria and their hosts. However, the specific role of OMVs in the pathogenic process of *M. haemolytica* remains poorly understood. To address this, we isolated OMVs from the *Mannheimia haemolytica* Type A5 strain (MH-5) using ultracentrifugation and subsequently characterized their secretory properties, protein composition, and immunogenicity through electron microscopy, liquid chromatography-tandem mass spectrometry (LC-MS/MS), and cellular experiments. The electron microscopy results indicated that the MH-5 strain secreted OMVs under natural growth conditions. Proteomic and bioinformatics analyses revealed that these OMVs contained 282 proteins, with significant enrichment in proteins related to immunity, iron metabolism, and catalytic activity. Cellular experiments demonstrated that, compared to the control group, the OMVs group exhibited a significant increase in the mRNA expression of IL-1β, IL-6, and TNF-α, with secretion levels increasing in a dose-dependent manner, thereby enhancing the inflammatory response. These findings lay the groundwork for further exploration of the role of OMVs in the pathogenesis of *M. haemolytica* and provide insights for the development of effective vaccines and antibiotics against this pathogen.

## 1 Introduction

*Mannheimia haemolytica* is a major pathogen responsible for bovine respiratory disease (BRD) and bovine respiratory disease complex (BRDC) (Gharib Mombeni et al., 2021)., causing acute and fibrinous necrotizing pneumonia, mastitis in sheep, and septicemia in young cattle and sheep, significantly impacting livestock morbidity and mortality (Klima et al., 2020). This close relationship poses challenges for accurate clinical diagnosis and the development of effective therapeutic strategies (Snyder and Credille, 2020). The onset of *M. haemolytica* infection is often associated with mixed infections caused by other bacteria and viruses, as well as secondary infections, further complicating the diagnosis, prevention, and control of this pathogen (Amat et al., 2020). The contagious nature of *M. haemolytica* (Snyder and Credille, 2020) results in high infection, morbidity, and mortality rates. Given the global trade of cattle and sheep, *M. haemolytica* poses a significant risk for the widespread dissemination of the disease. In North America alone, *M. haemolytica* has caused economic losses amounting to billions of dollars in the beef cattle farming industry, inflicting substantial damage on the global agricultural sector (Noyes et al., 2015). *M. haemolytica* can be classified into 12 serotypes based on surface antigens, including A1, A2, A5–A9, A12–A14, A16, and A17, with A1, A2, and A6 being the most prevalent, followed by A7, A9, A11, and A12. These serotypes primarily cause infections in cattle and sheep, with serotypes A1 and A6 being particularly responsible for bovine pneumonia (Crouch et al., 2012). However, serotypes A5, A6, and A7 have also been reported to cause disease in animals (Rice et al., 2007). The diversity of *M. haemolytica* serotypes poses a significant challenge to the control and prevention of bovine respiratory disease. Several key virulence factors have been identified in *M. haemolytica* including: Leukotoxin (LKT), which is specifically cytotoxic to ruminant leukocytes; Lipopolysaccharide (LPS), inducing inflammatory cytokine responses; Outer membrane proteins (OMPs), stimulating host immune responses; Adhesins, mediating bacterial colonization; Fimbriae (or pili), essential for bacterial adherence and invasion; Glycoproteases and neuraminidase, contributing to tissue damage and immune evasion (Fábio Pereira Leivas et al., 2003).

Many Gram-negative bacteria, including *M. haemolytica*, *Escherichia coli* (*E. coli*), *Salmonella enterica serovar Typhimurium* (*S.* Typhimurium), *Shigella flexneri* (*S. flexneri*), and *Helicobacter pylori* (*H. pylori*), are capable of producing nanoparticles with a spherical shape, encapsulated within a lipid bilayer, and typically ranging in size from 20 to 250 nm. These structures are known as outer membrane vesicles (OMVs) (MacNair and Tan, 2023). OMVs derived from Gram-negative bacteria typically arise from the explosive lysis of cells, which is often induced by vesicles or endolysins extruding from the outer membrane. These vesicles contain a variety of components similar to those found in the parental bacteria, including lipopolysaccharide (LPS), lipoproteins, peptidoglycans, DNA, and RNA (Ellis and Kuehn, 2010). Recently, some researchers have proposed that OMVs derived from Gram-negative bacteria do not conform to the traditional six secretion systems and are often considered a distinct secretion system, referred to as the type zero secretion system (T0SS) (Guerrero-Mandujano et al., 2017). OMVs, unlike other secretion mechanisms, enable bacteria to secrete insoluble molecules that bind to soluble substances, allowing enzymes to be recruited, hidden, and targeted for transport to distant sites. OMVs facilitate bacterial communication, enabling interactions with other bacteria, the environment, and bacterial communities, thereby contributing to the maintenance of environmental homeostasis. Additionally, OMVs play roles in biofilm formation and the dissemination of virulence factors, enhancing bacterial pathogenicity (Begić and Josić, 2020). For example, OMVs derived from *Porphyromonas gingivalis* and *Actinobacillus actinomycetemcomitans* enhance the adherence capabilities of various bacteria to host cells. Similarly, OMVs from *E. coli* and *dysentery bacilli* serve as carriers for toxins such as ClyA and Shiga toxins, respectively. *S.* Typhimurium, upon invading host cells, releases OMVs containing lipopolysaccharide (LPS) and other altered surface antigens, which can evade immune detection (Cui et al., 2022). However, relatively few studies have focused on the role of *M. haemolytica*-derived OMVs in pathogenesis. OMVs significantly enhance bacterial survival and pathogenicity by delivering toxins, LPS, DNA, RNA, small molecule compounds, and metal ions, among other components. This has sparked increasing interest in their roles in host colonization and disease pathogenesis, highlighting their potential for further research.

In this study, we have isolated and purified OMVs derived from the MH-5 strain. Based on previous studies (Sahlu et al., 2013), we hypothesize that OMVs play a crucial role in the MH-5 host infection process. Subsequently, we conducted a proteomic analysis and evaluated the immunogenicity of the OMVs to investigate their role in the pathogenicity of the MH-5 strain. First, the diameter and shape of OMVs were characterized using transmission electron microscopy and nano tracking. The protein components of MH-5-derived OMVs were analyzed using LC/MS/MS to elucidate the biological functions of individual proteins. The purified OMVs were used to assess their toxic effects on mouse macrophages (RAW264.7) at various concentrations, as well as their impact on cytokine expression. The findings provide a solid foundation for exploring the role of OMVs in the pathogenic mechanisms of MH-5.

## 2 Materials and methods

### 2.1 Culture of bacteria and cells

In 2022, a *Mannheimia haemolytica* strain (designated as MH-5) was isolated from the lungs of diseased sheep on a farm in Luoyang, Henan Province, China. The MH-5 strain was inoculated onto Tryptic Soy Peptone Agar (TSA) medium (ABXING Biotechnology Co., Ltd., Beijing, China) and incubated at 37°C for 12 to 24 h. Single colonies were carefully isolated and transferred to trypticase-soy peptone broth (TSB) medium (ABXING Biotechnology Co. Ltd., Beijing, China). The bacterial cultures were then incubated in a constant-temperature shaker at 37°C for 12 h. The bacterial optical density at 600 nm (OD_600_) was determined using a spectrophotometer. When the OD_600nm_ reaches 0.5 to 0.6, the bacterial solution is extracted directly and mixed with an equal volume of 50% glycerol and stored at –80°C for future use.

This study employed the mouse macrophage cell line RAW 264.7, obtained from (Punosai Biotechnology Co., Ltd., in Wuhan, China). RAW264.7 cells were cultured in complete DMEM medium (Cytiva Biotechnology Co., Ltd., Shanghai, China) supplemented with 10% fetal bovine serum. The cells were incubated at 37°C in a humidified atmosphere containing 5% CO_2_. Serum-free DMEM was used when necessary to minimize interference from exosomes and other proteins present in fetal bovine serum (Ahmed et al., 2021).

### 2.2 Extraction and purification of outer membrane vesicles of MH-5

OMVs were extracted with reference to the relevant literature (Xu, 2018). After MH-5 was identified by PCR without error, it was transferred to TSB liquid medium at a ratio of 1:100 for overnight incubation (12–14 h at approximately 2×10^8^ CFU). The colonies were washed three times with sterile phosphate buffer (PBS, 0.1 M, pH 7.3). After washing, the colonies were resuspended with PBS and evenly spread onto TSA solid Petri dishes, followed by incubation at 37°C for 72 h. Cotton swabs were used to collect bacteria from the Petri dishes, which were then suspended in 30 mL of sterile PBS. The bacterial suspension was centrifuged at 10,000 × g for 30 min. The samples were incubated in a 56°C water bath for 30 min with vigorous vortex mixing. The bacterial suspension was then centrifuged at 10,000 × g for 30 min, and the supernatant was collected after centrifugation. The supernatant was filtered through a 0.22 μm filter membrane. 100 μL of the filtered supernatant was coated onto TSA dishes and incubated at 37°C for 72 h. The sterility of the filtered supernatant was confirmed. The filtered supernatant, confirmed to be sterile, was centrifuged at 100,000 × g for 2 h at 4°C. The precipitates were washed twice with 1 mL of sterile PBS, and then the precipitates (OMVs) were suspended in 1 mL of sterile PBS. The total protein concentration of OMVs was determined using a BCA protein kit. OMVs were divided into 0.5 mL aliquots and stored at –80°C.

Purification of OMVs was achieved using an ultra-pure size exclusion chromatography column (SuperEV). The bottom cover of the column was carefully removed, and PBS above the sieve plate was aspirated using a pipette. Subsequently, 4 mL of the concentrated OMVs solution was added above the sieve plate. A 15 mL centrifuge tube was positioned beneath the column to collect the first fraction. After all samples were introduced into the sieve plate, 10.5 mL of PBS was added. The process was considered complete once all liquid had drained into the underside of the sieve plate and no liquid was observed flowing out of the outlet. Fraction 1, representing the void volume of approximately 14.5 mL, was devoid of OMVs. Elution was then initiated by the addition of PBS, and the resulting fraction was collected in a 2 mL centrifuge tube. PBS was added in 2 mL increments, with collection ceasing once all eluate had reached the sieve plate and no liquid emerged from the outlet. The next 2 mL of PBS was then added for collection. Each fraction was collected in volumes of 2 mL.

### 2.3 Transmission electron microscopy observations

Extracted OMVs are dissolved in 50–100 µL of 2% paraformaldehyde solution (can be stored at 4°C for up to one week). Took 5–10 µL of OMVs solution and added it to the Formvar-carbon carrier copper grid. Add 100 µL of PBS to the sealing membrane and wash the copper mesh (Formvar membrane side down) by placing it on a droplet of PBS using forceps. Place the copper mesh on a 50 µL 1% glutaraldehyde droplet for 5 min. Then wash the copper mesh in 100 µL ddH_2_O for 2 min (8 washes). Place the copper mesh on a 50 µL droplet of uranyl oxalate (pH 7.0) for 5 min. Then place the copper mesh on a 50 µL droplet of methylcellulose for 10 min and manipulate it on ice. Air dried for 5–10 min, placed the copper mesh in a sample box, and took electron micrographs at 80 kV.

### 2.4 Sodium dodecyl sulfate-polyacrylamide gel electrophoresis

OMVs proteins were quantified using a BCA protein assay kit (Beyotime Biotechnology, P0010S). OMVs proteins from the MH-5 were analyzed by 10% SDS-PAGE, followed by staining with Coomassie Brilliant Blue and decolorization with glacial acetic acid. The gel was loaded with protein molecular weight standards ranging from 15 to 150 kDa.

### 2.5 Liquid chromatography tandem mass spectrometry

For in-gel tryptic digestion, gel pieces were decolorized in 50 mM NH_4_HCO_3_ in 50% acetonitrile (v/v) until clear. Gel pieces were dehydrated with 100 μL of 100% acetonitrile for 5 min, the liquid removed, and the gel pieces rehydrated in 10 mM dithiothreitol and incubated at 56°C for 60 min. Gel pieces were again dehydrated in 100% acetonitrile, liquid was removed, and gel pieces were rehydrated with 50 mM iodoacetamide. Samples were incubated at room temperature, in the dark for 45 min. Gel pieces were washed with 50 mM NH_4_HCO_3_ and dehydrated with 100% acetonitrile. Gel pieces were rehydrated with 10 ng/μL trypsin resuspended in 50 mM NH_4_HC0_3_ and 5 mM CaCl_2_ on ice for 10 min. Excess liquid was removed and gel pieces were digested with trypsin at 37°C overnight. Peptides were extracted with 50% acetonitrile/5% formic acid, followed by 100% acetonitrile. Peptides were dried to completion and resuspended in 2% acetonitrile/0.1% formic acid.

The tryptic peptides were dissolved in 0.1% formic acid (solvent A), directly loaded onto a home-made reversed-phase analytical column (15-cm length, 75 μm i.d.).The gradient was comprised of an increase from 5% to 34% solvent B (0.1% formic acid in 80% acetonitrile) over 40 min, 34% to 38% in 5 min, 38% to 90% in 10 min and then holding at 90% for the last 10 min, all at a constant flow rate of 300 nL/min on an EASY-nLC 1200 UPLC system.

The peptides were subjected to NSI source followed by tandem mass spectrometry (MS/MS) in Thermo Scientific Orbitrap Exploris 480 coupled online to the UPLC. The electrospray voltage applied was 2.0 kV, The m/z scan range was 350 to 1800 for full scan, and intact peptides were detected in the Orbitrap at a resolution of 70,000. Peptides were then selected for MS/MS using NCE setting as 28 and the fragments were detected in the Orbitrap at a resolution of 17,500. A data-dependent procedure that alternated between one MS scan followed by 20 MS/MS scans with 15.0 s dynamic exclusion. Automatic gain control (AGC) was set at 5E4.

### 2.6 Bioinformatics analysis of outer membrane vesicle proteins

The resulting MS/MS data were processed using Proteome Discoverer 2.4. Tandem mass spectra were searched against Swissprot_Human. Trypsin/P was specified as cleavage enzyme. Mass error was set to 10 ppm for precursor ions and 0.02 Da for-fragment ions. Carbamidomethyl on Cys was specified as fixed modification. Upload the data obtained from protein profiling to the web site “https://www.uniprot.org/” for comparative analysis.

### 2.7 Mouse macrophage RAW 264.7 viability assay

Morphological characterization of RAW264.7 murine macrophages exposed to a concentration gradient of OMVs for 24 hours was conducted via bright-field microscopy (40 × magnification), with systematic image acquisition to enable comparative analysis of cellular structural changes.

Cell viability was determined using a cell counting kit (CCK-8) (Beyotime Biotechnology, Shang hai, China). OMVs were prepared at 0.78 μg/mL, 1.56 μg/mL, 3.12 μg/mL, 6.25 μg/mL, 12.5 μg/mL, and 25 μg/mL, respectively. Mouse macrophage RAW264.7 was inoculated into 96-well plates at a cell volume of 5 × 10^5^ cells/well and incubated for 24 h according to step **1.1.** Different concentrations of OMVs were added to the cultured cell stock solution, serving as the positive group. Sterile DMEM was used as the blank group, while fresh macrophage RAW264.7 cells constituted the control group. Each group consisted of five replicates. Incubate for 24 h under the same conditions. At the conclusion of the incubation cycle, the cell culture medium was replaced with 100 μL of fresh cell culture medium, to which 10 μL of CCK-8 reagent was added. The cells were then incubated for an additional 2 h. Finally, absorbance was measured at OD_450_ nm using an enzyme marker (Multiskan EX, Thermo Fisher Scientific, USA). The formula was calculated as: cell viability = (absorbance sample - absorbance blank)/(absorbance control - absorbance blank) x 100%.

### 2.8 Fluorescence quantitative PCR test

RAW264.7 cells were divided into two groups: those not stimulated with OMVs (control) and those stimulated with two different concentrations of OMVs, as determined by CCK8 assays. Total cellular RNA was extracted following the kit’s protocol, reverse-transcribed into cDNA, and the reaction system was set up according to the kit’s instructions for RT-qPCR (Takara Bio, Dalian, China). The expression of cell-associated inflammatory cytokines, including IL-6, IL-1β, TNF-α, INF-γ, and IL-18, was quantified relative to β-actin. The primer sequences are listed in **Table 1**.

**Table 1:** Primers for inflammatory factors.

Targeting genes	Primer sequence(5’→3’)	Accession no.	Reference
*IL-6*	F:TGATGGATGCTACCAAACTGGA R:TGTGACTCCAGCTTATCTCTTGG	NM_001314054.1	(Mora et al., 2024)
*IL-1β*	F:TGCCACCTTTTGACAGTGATG R:TGATGTGCTGCTGCGAGATT	NM_008361.4	(McClure et al., 2024)
*TNF-α*	F:AGCCGATGGGTTGTACCTTG R:AGTACTTGGGCAGATTGACCTC	NM_001278601.1	(McClure et al., 2024)
*IFN-γ*	F:GAACGCTACACACTGCATCT R:TCAGCAGCGACTCCTTTTCC	NM_008337.4	(Lin et al., 2024)
*IL-18*	F:ATGGCTGCCATGTCAGAAGA R:GGCAAGCAAGAAAGTGTCCTTC	NM_001357221	(Zhao et al., 2024)

### 2.9 Statistical analysis

All experiments were replicated three times for each sample, and the results are presented as the mean ± standard deviation (SD). Statistical analyses and comparisons were conducted using SPSS 24.0 (IBM, USA) and GraphPad Prism version 9.4.1.681 (GraphPad Software, Inc., USA). One-way analysis of variance (ANOVA) was employed to assess the qPCR assay outcomes. (***) = *P* < *0.001*; (**) = *P* < *0.01*; (*) = *P* < *0.05*; (ns) = No significant difference.

## 3 Results

### 3.1 Purification and characterization of outer membrane vesicle proteins of MH-5

**Figure 1:**
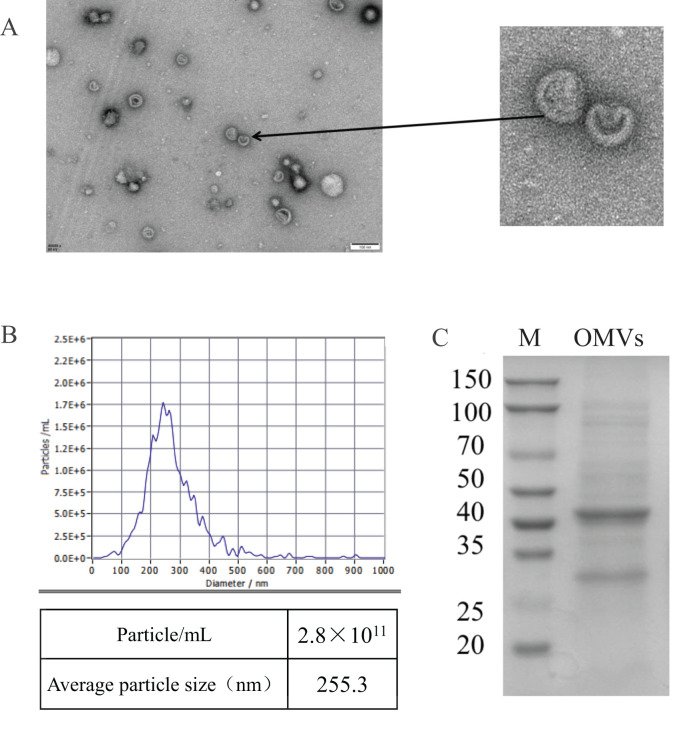
Characterization of OMVs of MH-5 strain. **(A)** Transmission electron microscopy images of MH-5 OMVs showing the size of OMVs, Scale bar: 100 nm, **(B)** OMVs nanoparticle size tracking analysis (NTA), **(C)** Comparison of protein composition of different parts of MH-5 by SDS-PAGE.

OMVs were purified using SuperEV and subsequently examined under transmission electron microscopy. The electron microscopy findings indicated that the OMVs appeared as double-layered, spherical vesicles, consistent with the ‘Teatray shaped’ characteristics (**Figures 1A, B**). The average diameter of OMVs was 255.3 nm and the particle number was 2.8 × 10^11^/mL. The results show that the OMVs particle size distribution is in a narrow range and the concentration is high.

### 3.2 Proteomic analysis of outer membrane vesicles of MH-5

**Figure 2:**
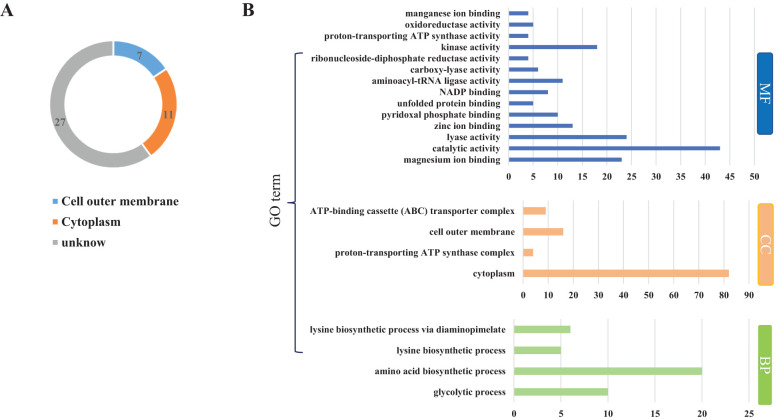
Distribution of major proteins and GO analysis of OMVs of MH-5 strain. **(A)**: Distribution of major proteins, **(B)**: GO function annotation analysis. Molecular Function (GOMF): gene molecular function, Cellular Component (GOCC): cellular component, Biological Process (GOBP): biological process function.

**Figure 3:**
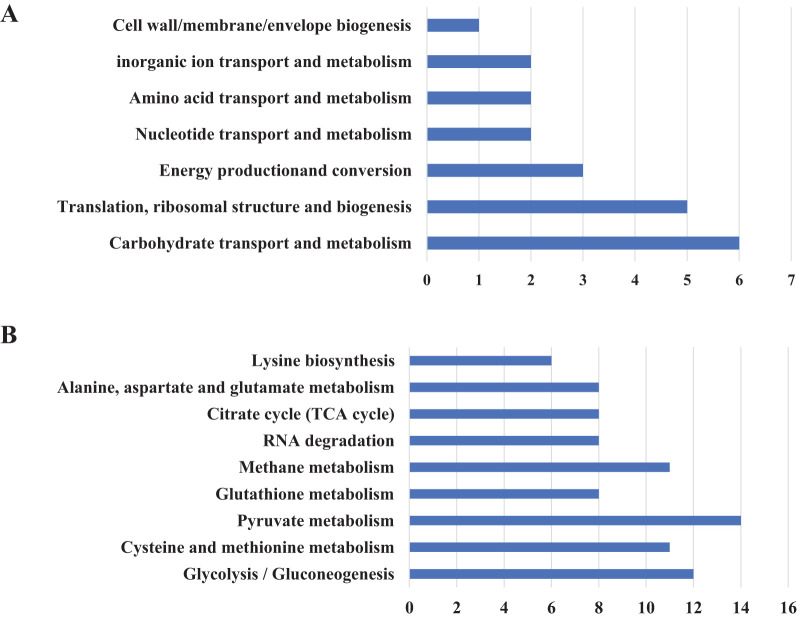
Overview of proteome identification of MH-5-derived OMVs. **(A)** COG analysis, **(B)** KEGG prediction.

To investigate the protein composition of OMVs derived from MH-5, LC-MS/MS analysis was employed to identify the protein content, resulting in the detection of a total of 282 proteins. All proteins identified were first subcellularly localized: cytoplasmic (n = 82), extracellular membrane (n = 16) (**Figure 2B**). OMVs were enriched in membrane proteins (n = 98), suggesting that MH-5 outer membrane proteins were highly enriched in OMVs. Additionally, the most prominent proteins analyzed from 282 proteins, which were associated with immunogenicity, virulence factors, and iron uptake, were subcellularly localized to the extracellular membrane (n = 7), cytoplasm (n = 27), and unknown (n = 27) (**Figure 2A**). In addition, functional annotation analysis using the gene ontology (GO), cluster of orthologous groups of proteins (COG) and Kyoto encyclopedia of genes and genomes (KEGG) pathways was conducted for all identified proteins (**Figure 2B**). The genetic molecular functions (GOMF) of OMVs proteins were primarily centered around catalytic activity (n = 43), with a significant number also associated with layase activity (n = 24) and magnesium ion binding (n = 23). The biological processes’ function (GOBP) of OMVs proteins is primarily involved in amino acid biosynthesis (n = 20), and to a lesser extent, in glycolytic processes (n = 10). An annotated analysis of the protein clusters of COG functions of OMVs (**Figure 3A**) revealed that the most frequently associated functions were related to carbohydrate transport and metabolism (n = 6), followed by functions related to protein synthesis and translation, ribosome structure, and biogenesis (n = 5). KEGG annotation pathway analysis (**Figure 3B**), with pyruvate metabolism being the most abundant (n = 14). The findings indicate that OMVs are crucial for the survival of MH-5 in the external environment, its invasion of the host, colonization, and infection processes.

### 3.3 Outer membrane vesicles carry immunomodulatory and virulence-related proteins

We quantified the extracted OMVs using a BCA Protein Concentration Assay Kit. The measured concentration was 1 μg/μL, and 20 μL of the sample was used for SDS-PAGE analysis. The results showed that OMVs contained a large number of proteins associated with *Mannheimia haemolytica* (MH-5). (**Figure 1C**). Based on the proteomics results of OMVs, we found that they were enriched in proteins related to immunomodulation, virulence factors, iron uptake, and resistance genes (**Table 2**). For example, proteins associated with immunogenicity are OmpA1 (A0A3S5B1Z0), OmpH (A0A448TCS4), Outer membrane pore protein (A0A448T9U9), and TufA2 (A0A249A2N2). These results suggest that OMVs can induce some degree of protective immunity. Proteins related to virulence factor are LktA (A0A3S4XCH7), TalB (A0A448TA02), and MetE1 (A0A448TA65). Bacteria regulate the genes associated with their energy metabolism in response to the host’s internal environment to adapt for survival. Simultaneously, they utilize host nutrients to generate energy, which is essential for initiating their subsequent pathogenic processes, including bacterial migration, colonization, growth, adhesion, and others, within the host environment (François et al., 2021). For example, glycolysis-related proteins are PykA (A0A3S4XQ93), GpmA (A0A3S5BBG0), AceE (A0A448TCF5), FbaA (A0A248ZXM7), EnO (A0A249A1S0), PckA (A0A3S4YI01), PgK (A0A3S5B6R5), AceF (A0A3S5F3L0). Iron is an essential nutrient for nearly all bacteria, and pathogenic strains have evolved various mechanisms to acquire sufficient free iron from their hosts. These mechanisms include the expression of surface-exposed proteins that bind to iron carriers, blood carriers, or host molecules, as detailed by reference (Kunli et al., 2021). Examples include Tbp1 (A0A3S4XZ36), HbpA1 (A0A448T6M1), and TbpB (A0A448TDZ6). Drug resistance is one of the reasons why *M. haemolytica* is difficult to control. Several relevant drug resistance-associated proteins, LpoA (A0A3S4XT40), RpoC (A0A3S5B8E1), IleS (A0A448T988), PnP (A0A448TBI9),FabB (A0A248ZXQ9), and LamB (A0A3S4X0U9), were identified from OMVs.

**Table 2:** Major proteins identified from *Mannheimia haemolytica* MH-5 OMVs using LC-MS/MS.

Protein_ID	MW [kDa]	Gene Name	Description	Molecular Function
A0A3S5B1Z0	39.2	*ompA_1*	Outer membrane protein A	Cell outer membrane
A0A448TCS4	39.6	*ompH*	Outer membrane protein H	Cell outer membrane
A0A448T9U9	41	*NCTC10643_00961*	Outer membrane protein (Porin)	Cell outer membrane
A0A448T7C8	56	*glpK*	Glycerol kinase	Kinase activity
A0A3S4XCH7	102.1	*lktA*	Leukotoxin	Virulence factor
A0A3S4XX79	57.6	*groL*	Chaperonin GroEL	Cytoplasm
A0A248ZXR5	26	*deoD*	Purine nucleoside phosphorylase DeoD-type	Glycolytic process
A0A3S4XDB1	58	*gsiB*	Glutathione-binding protein	ATP-binding cassette (ABC) transporter complex
A0A3S4XT40	63.3	*lpoA*	Penicillin-binding protein activator	Magnesium ion binding
A0A3S4XQ93	51.6	*pykA*	Pyruvate kinase	Catalytic activity
A0A249A2N2	43.3	*tufA_2*	Elongation factor Tu	Cytoplasm
A0A3S5F331	77	*fusA*	Elongation factor G	Cytoplasm
A0A3S5BBG0	26	*gpmA*	2,3-bisphosphoglycerate-dependent phosphoglycerate mutase	Glycolytic process
A0A448T9Y7	33.3	*cysK*	Cysteine synthase	Amino acid biosynthetic process
A0A3S4XCM8	43.4	*aspC*	Aminotransferase	Catalytic activity
A0A3S4X9I0	59.8	*NCTC10643_00522*	Probable phosphomannomutase	Magnesium ion binding
A0A448TCF5	98.8	*aceE*	Pyruvate dehydrogenase E1 component	Glycolytic process
A0A3S5B8E1	157.9	*rpoC*	DNA-directed RNA polymerase subunit beta	Zinc ion binding
A0A448T7S1	67.3	*glmS*	Glutamine–fructose-6-phosphate aminotransferase [isomerizing]	Cytoplasm
A0A248ZXM7	39.1	*fbaA*	Fructose-bisphosphate aldolase	Glycolytic process
A0A3S4XZ36	107.3	*tbp1*	Transferrin-binding protein 1	Cell outer membrane
A0A249A1S0	46.1	*eno*	Enolase	Glycolytic process
A0A3S4YI01	59.4	*pckA*	Phosphoenolpyruvate carboxykinase	Cytoplasm
A0A448TA65	43.5	*metE_1*	5-methyltetrahydropteroyltriglutamate-homocysteine methyltransferase	Zinc ion binding
Q51848	40.1	*potD*	Putrescine-binding periplasmic protein	Amino acid transport and metabolism
A0A448TEW5	33.8	*mdh*	Malate dehydrogenase	Catalytic activity
A0A3S5B6R5	41.5	*pgk*	Phosphoglycerate kinase	Glycolytic process
A0A249A138	66.2	*ilvD*	Dihydroxy-acid dehydratase	Amino acid biosynthetic process
A0A448TC90	39.9	*serC*	Phosphoserine aminotransferase	Amino acid biosynthetic process
A0A249A0J9	35.7	*gapA_2*	Glyceraldehyde-3-phosphate dehydrogenase	NADP binding
A0A448TA02	35	*talB*	Transaldolase	Cytoplasm
A0A3S4XLX6	54	*mmsA*	Methylmalonate-semialdehyde dehydrogenase [acylating]	Oxidoreductase activity
A0A448T988	105.5	*ileS*	Isoleucine-tRNA ligase	Cytoplasm
A0A1D2Q7B1	45.4	*iscS*	Cysteine desulfurase	Cytoplasm
A0A448TBI9	77.6	*pnp*	Polyribonucleotide nucleotidyltransferase	Cytoplasm
A0A248ZXQ9	42.6	*fabB*	3-oxoacyl-[acyl-carrier-protein] synthase 1	Cytoplasm
A0A3S4XDP7	86.7	*pflB*	Formate acetyltransferase	Cytoplasm
A0A448T6M1	59.4	*hbpA_1*	Hemin-binding lipoprotein	ATP-binding cassette (ABC) transporter complex
A0A249A2P6	35.6	*mglB*	D-galactose/methyl-galactoside binding periplasmic protein	ATP-binding cassette (ABC) transporter complex
A0A3S5BD43	43.4	*malE*	Maltodextrin-binding protein	Pyridoxal phosphate binding
A0A3S5F3L0	66.6	*aceF*	Acetyltransferase component of pyruvate dehydrogenase complex	Glycolytic process
A0A3S4YIG4	49	*degP*	Periplasmic serine endoprotease	Cell outer membrane
A0A448T8W8	46.4	*thrC*	Threonine synthase	Lyase activity
A0A3S4X0U9	47.3	*lamB*	Maltose-inducible porin	Cell outer membrane
A0A448TDZ6	63.4	*tbpB*	Transferrin-binding protein B	Cell outer membrane

### 3.4 Effect of outer membrane vesicles on survival of mouse macrophage RAW264.7

**Figure 4:**
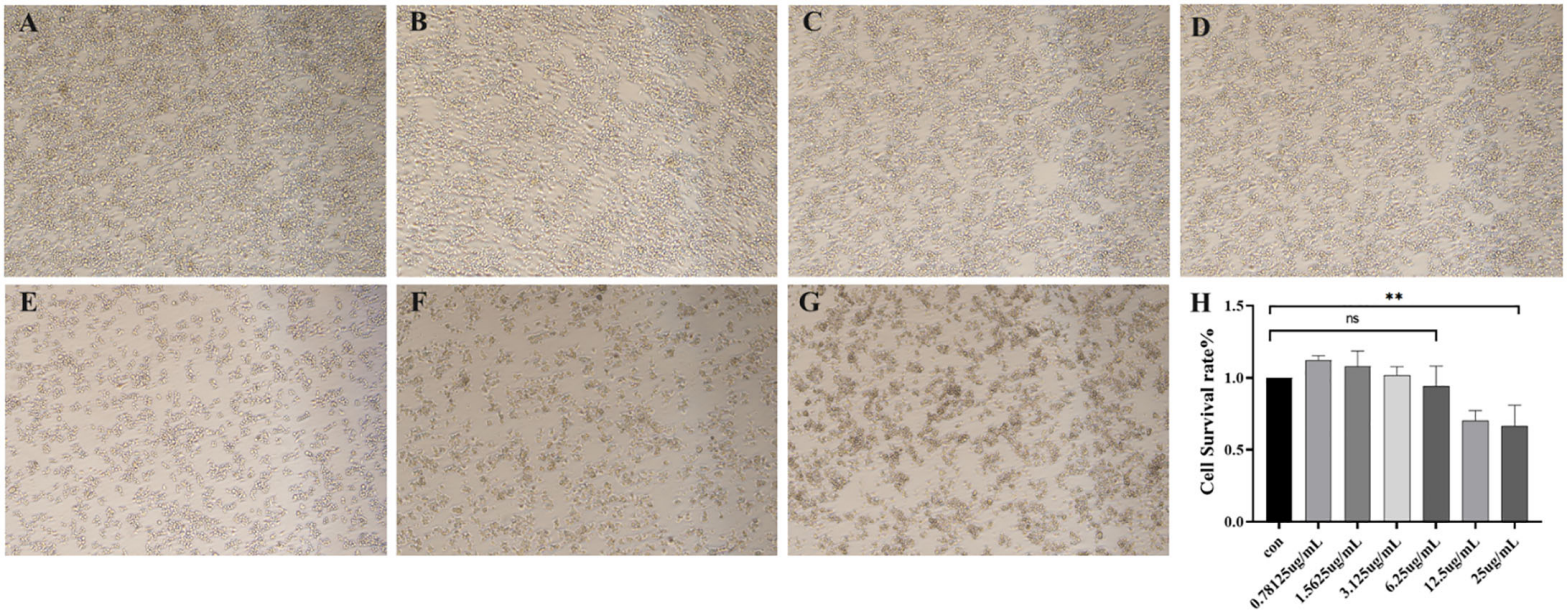
**(A–G)** Morphological assessment of RAW264.7 murine macrophages after 24 h of exposure to outer membrane vesicles (OMVs) at different concentrations (40 x) **(A)** Cell state in normal culture **(B)** Cellular morphological changes induced by OMVs at 0.78125 μg/mL **(C)** Cellular morphological changes induced by OMVs at 1.5625 μg/mL **(D)** Cellular morphological changes induced by OMVs at 3.125 μg/mL **(E)** Cellular morphological changes induced by OMVs at 6.25 μg/mL **(F)** Cellular morphological changes induced by OMVs at 12.5 μg/mL **(G)** Cellular morphological changes induced by OMVs at 25 μg/mL **(H)** OMVs action RAW264.7 survival at different concentrations. *P* > 0.05 (ns); *P* < 0.01 (**), (ns) = No significant difference.

Viewed through a microscope, the results demonstrated that control group murine macrophages maintained a round, translucent morphology with homogeneous cytoplasmic density and well-defined cellular margins. In contrast, OMV-treated RAW264.7 cells exhibited dose-dependent morphological alterations, including prominent vacuolization, membrane crumpling, and significant reduction in cellular density compared to untreated controls (**Figures 4A–G**).

The cytotoxic effects of OMVs, which harbor toxic components like endotoxin, were investigated by assessing their impact on mouse macrophages at various concentrations (**Figure 4H**). At concentrations of 0.78 μg/mL, 1.56 μg/mL, and 3.12 μg/mL, the OMVs did not exhibit significant cytotoxicity compared to the control group, indicating no notable cell damage. However, at a concentration of 6.25 μg/mL, there was a slight increase in cytotoxicity. Further increases to 12.5 μg/mL and 25 μg/mL resulted in severe cytotoxicity.

### 3.5 Inflammatory response of the outer membrane vesicle of MH-5

**Figure 5:**
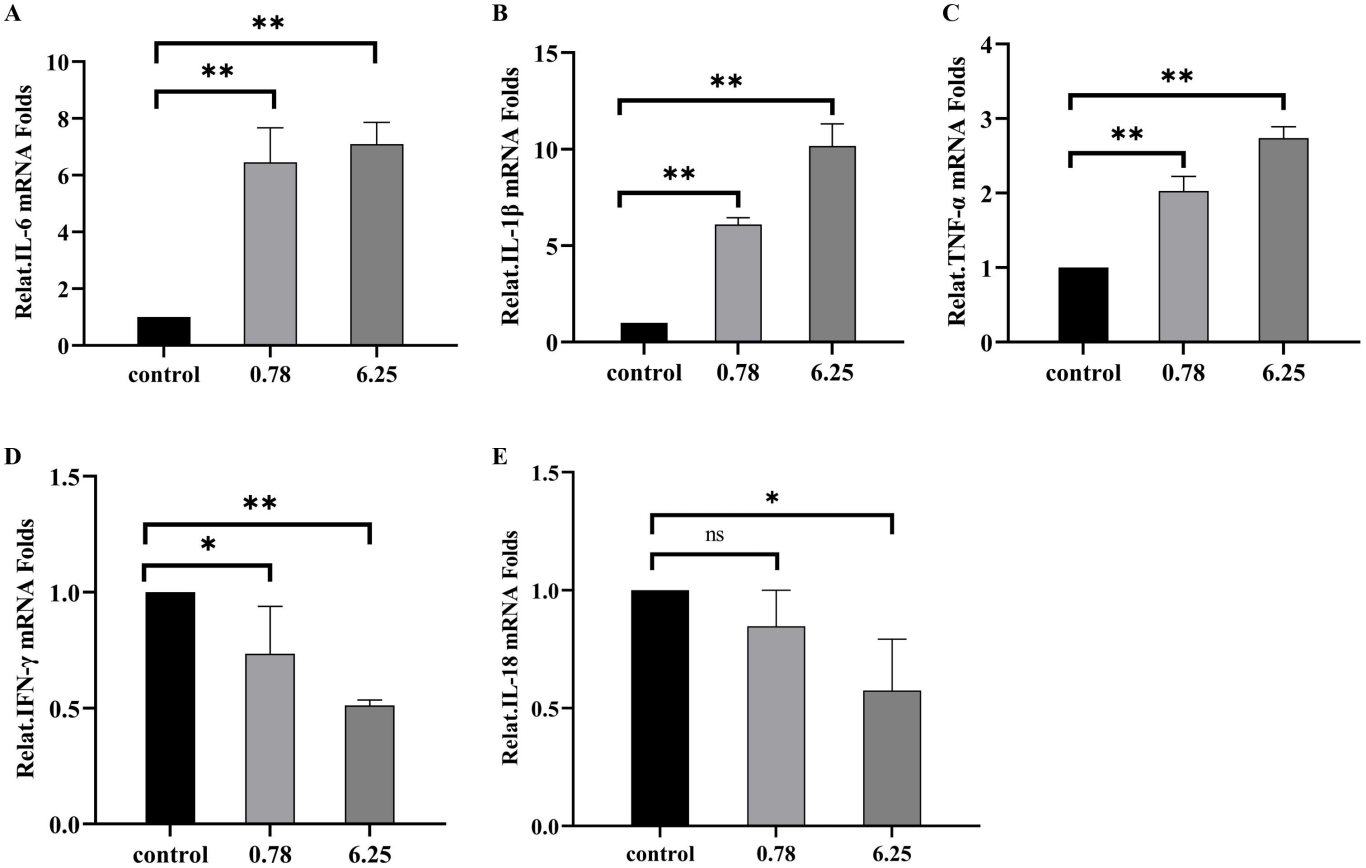
*Mannheimia haemolytica* MH-5 strain OMVs stimulate inflammatory responses in mouse macrophages (RAW264.7) **(A)** IL-6 mRNA expression, **(B)** IL-1β mRNA expression, **(C)** TNF-α mRNA expression, **(D)** INF-γ mRNA expression, **(E)** IL-18 mRNA expression. *P* > 0.05 (ns); *P <* 0.05 (*); *P <* 0.01 (**).

To assess the capacity of MH-5 OMVs to induce the transcription and secretion of various inflammatory cytokines in mouse monocyte-derived macrophages (RAW264.7), two OMV concentrations were employed: 0.78 μg/mL and 6.25 μg/mL. The expression and secretion of IL-1β, IL-6, and TNF-α mRNA were significantly enhanced in a dose-dependent fashion following OMV infection. It was demonstrated that OMVs could stimulate the RAW264.7 cells to exhibit Th1 (IL-6, TNF-α) and Th17 (IL-1β) cell immunity types in mouse macrophages (**Figures 5A–C**). In contrast, the expression of INF-γ and IL-18 mRNA was found to be lower than in the control group (**Figures 5D, E**).

## 4 Discussion

*M. haemolytica* is a significant pathogen in the livestock farming industry, causing respiratory diseases in cattle and sheep, as well as acute sepsis in newborn calves (Alhamami et al., 2021). Recent studies have also indicated that *M. haemolytica* can cause mastitis in sheep and may be transmitted to lambs through lactation (Lida et al., 2011, 2016), thereby complicating prevention and control efforts against this pathogen. Traditional treatments, such as antibiotics and vaccines, have been found to have significant drawbacks over the years. These include the emergence of bacterial resistance and the lack of 100% vaccine protection, both domestically and internationally (Christian et al., 2020). These challenges have caused substantial economic losses to the global farming industry (Jyoti et al., 2015). Therefore, a comprehensive understanding of *M. haemolytica’s* pathogenic mechanisms is crucial for preventing and controlling its spread. Existing research has demonstrated that OMVs, as an autonomous secretion system in Gram-negative bacteria, are involved in various biological processes, including population sensing, dissemination of antibiotic resistance genes, and release of toxins and virulence determinants (Sandro et al., 2013). These interactions, both among bacteria and between bacteria and their hosts (Maria and Richard, 2015), contribute to the emergence of bacterial diseases that pose significant challenges for prevention and control. Currently, research on OMVs produced by *M. haemolytica* remains limited, highlighting the need for further exploration in this area. In this study, we successfully extracted OMVs from strain MH-5 using ultracentrifugation and characterized them via transmission electron microscopy, revealing a substantial population of round and oval vesicle-like structures with double membranes. Particle size analysis revealed that the average size of MH-5 OMVs was approximately 255.3 nm, with a concentration of 2.8 × 10^11^ units/mL. These findings corroborate previous literature reports (Mirosław et al., 2020), indicating that MH-5 can secrete OMVs into the extracellular environment during *in vitro* cultivation.

Proteomic analysis of the 282 identified proteins (**Figure 2B**) revealed that cytoplasmic proteins constituted over half of the total, with OMPs being the next most abundant. However, studies on other Gram-negative bacteria have shown that outer membrane (and extracellular) proteins are the major components of OMVs (Altindis et al., 2014; Veith et al., 2014). This discrepancy is unlikely to be due to contamination, as the purification methods used were virtually identical. Rather, the variation may result from differences in biochemical profiles among bacterial species and culture conditions (Pérez-Cruz et al., 2015; Liu et al., 2019). In addition, GO, COG, and KEGG pathway analyses were performed on all identified proteins. The GOMF proteins in OMVs are primarily involved in various functions, including catalytic activity, lyase activity, and magnesium ion binding. GOBP analysis indicated that OMV proteins are predominantly associated with amino acid biosynthesis, glycolytic processes, diaminopimelate metabolism, and lysine biosynthesis. COG analysis revealed that most OMV proteins are involved in carbohydrate transport and metabolism. KEGG pathway analysis further highlighted the involvement of OMV proteins in pathways such as pyruvate metabolism, glycolysis/gluconeogenesis, cysteine and methionine metabolism, and methane metabolism, with the latter containing the highest protein concentration. The findings suggest that OMVs play a crucial role in helping MH-5 adapt to challenging external environments, penetrate host defenses, establish colonization, and cause infection.

Many studies have demonstrated that OMVs isolated from pathogenic bacteria are highly protective against pathogenic infections (Qiong et al., 2017; Michael et al., 2018; Solanki et al., 2021). Proteomic analysis of MH-5 OMVs revealed that they are enriched with numerous proteins related to immunogenicity, virulence factors, and iron uptake. These proteins included OmpA1, OmpH, LktA, TalB, TbpL, and HbpA1 (**Table 2**). Among these proteins, OmpA and OmpH are particularly significant immunogenic components of *M. haemolytica* (Cecilia et al., 2022; Sahlu et al., 2011). These proteins are highly conserved, species-specific, and surface-exposed. Previous studies have shown that immunizing cattle with recombinant *M. haemolytica* OmpA successfully induced high levels of antibodies (Sahlu et al., 2011). Another study found that incubating *M. haemolytica* with sera from rabbits immunized against OmpH significantly reduced bacterial adhesion to cells by 45%. Furthermore, OmpH was detected in bovine sera from animals with acute or chronic respiratory disease, indicating its *in vivo* expression and cross-reactivity with sera from rabbits infected with *Pasteurella multocida* (Cecilia et al., 2022). These findings suggest that OMVs could serve as a potential subunit vaccine against *M. haemolytica*. OMVs derived from the MH-5 strain carry numerous proteins linked to pathogenic mechanisms, suggesting their crucial role in MH-5 infection. LktA, a well-known virulence factor of *M. haemolytica*, damages ruminant leukocytes and alveoli by inducing the release of enzymes and oxygen free radicals. Additionally, LktA can trigger the release of pro-inflammatory mediators from the lungs, further exacerbating lung lesions (Dounia et al., 2021). Recent advancements have seen increased use of OMVs for expressing exogenous proteins, leveraging the inherent immunogenicity of ClyA to enhance immune responses to antigens (Zhuang et al., 2021). For example, fusing *Acinetobacter baumannii* Omp22 with ClyA from *E. coli* DH5α was used to immunize mice against *Acinetobacter baumannii*, resulting in higher survival rates upon pathogen challenge (Huang et al., 2016). This approach, which involves delivering *M. haemolytica* Lkt virulence factors via OMVs as a vaccine, offers promising avenues for future research (Guerrero-Mandujano et al., 2017).

*M. haemolytica* induces a range of inflammatory responses in the host; however, the inflammatory effects of OMVs on host cells remain poorly understood. In the present study, OMVs derived from the MH-5 strain exhibited mild toxicity towards RAW264.7 murine macrophages at a concentration of 6.25 μg/mL, while severe cytotoxicity was observed at 12.5 μg/mL, indicating a significant, dose-dependent increase in toxicity. Subsequently, qPCR analysis revealed that OMVs exerted inflammatory effects at various concentrations, significantly enhancing the expression and secretion of cytokines (IL-6, IL-1β, and TNF-α) in a dose-dependent manner. The findings suggested that OMVs stimulated RAW264.7 murine macrophages to trigger both Th1-associated (IL-6, TNF-α) and Th17-associated (IL-1β) immune responses (Oh Youn et al., 2013; Chatterjee and Chaudhuri, 2013). In summary, OMVs are a critical element of the inflammatory response triggered by MH-5 infection. Previous research (Sivapriya Kailasan et al., 2016) has demonstrated that OMVs induce inflammation primarily via LPS. Based on the experimental results of this study, the interaction between MH-5 OMVs and bovine macrophage cell lines is very meaningful and points the way to the next step of exploring the pathogenic mechanism of *M. haemolytica* more deeply. Specifically, OMVs serve as carriers that facilitate the translocation of LPS from the extracellular to the intracellular space during Gram-negative bacterial infections. By delivering LPS into the cytoplasm and binding to cellular receptors, OMVs initiate caspase-1 activation and cellular pyroptosis, underscoring the pivotal role of OMVs in bacterial infection. Therefore, exploring strategies to effectively neutralize LPS within OMVs is of great interest, aiming to enhance vaccine development and mitigate infection-induced damage in animals.

## 5 Conclusions

In summary, this paper presents a comprehensive study of the morphology and proteomics of OMVs from the clinical isolate MH-5. Analysis revealed that MH-5-derived OMVs contained 282 proteins, including those linked to immunogenicity, virulence factors, iron metabolism, glycolysis, and drug resistance. Cellular experiments further demonstrated that MH-5-derived OMVs are toxic to mouse macrophages (RAW264.7), inducing the production of pro-inflammatory cytokines IL-1β, IL-6, and TNF-α. These findings support the hypothesis that MH-5-derived OMVs contribute to the pathogenic mechanism of MH-5 during infection.

## Data Availability

Data are available via ProteomeXchange with identifier PXD064633.
