# Supplementary material for: Proteomic analysis of outer membrane vesicles derived from the type A5 Strain of Mannheimia haemolytica
Source: Front Cell Infect Microbiol. 2025 Jun 11;15:1578027. doi: 10.3389/fcimb.2025.1578027 (PMC12187688; doi:10.3389/fcimb.2025.1578027)
Supplement: Supplementary file 1 [file Table1.docx]

Supplementary Material

Table S1 proteins identified from OMVs of *Mannheimia haemolytica* MH-1 strain.

| \| \| **No.** \| **Accession** \| **Description** \| **Coverage [%]** \| **Peptides** \| **PSMs** \| **Unique Peptides** \| **AAs** \| **MW [kDa]** \| **calc. pI** \| **Score Sequest H** \| **Peptides** \| **Protein**  **Groups** \| \| --- \| --- \| --- \| --- \| --- \| --- \| --- \| --- \| --- \| --- \| --- \| --- \| --- \| \|  \| A0A3S5B1Z0 \| Outer membrane protein A OS=Mannheimia haemolytica OX=75985 GN=ompA_1 PE=3 SV=1 \| 65 \| 15 \| 147 \| 15 \| 367 \| 39.2 \| 9 \| 468.89 \| 15 \| 1 \| \|  \| A0A448TCS4 \| 37 kDa outer membrane protein OS=Mannheimia haemolytica OX=75985 GN=ompH PE=4 SV=1 \| 40 \| 13 \| 149 \| 13 \| 354 \| 39.6 \| 9 \| 412.12 \| 13 \| 1 \| \|  \| A0A448T9U9 \| Outer membrane protein (Porin) OS=Mannheimia haemolytica OX=75985 GN=NCTC10643_00961 PE=4 SV=1 \| 34 \| 11 \| 48 \| 11 \| 364 \| 41 \| 9.52 \| 118.51 \| 11 \| 1 \| \|  \| A0A448T7C8 \| Glycerol kinase OS=Mannheimia haemolytica OX=75985 GN=glpK PE=3 SV=1 \| 26 \| 8 \| 29 \| 8 \| 503 \| 56 \| 5.96 \| 88.31 \| 8 \| 1 \| \|  \| A0A3S4XCH7 \| Leukotoxin OS=Mannheimia haemolytica OX=75985 GN=lktA PE=3 SV=1 \| 17 \| 13 \| 26 \| 13 \| 953 \| 102.1 \| 6.34 \| 79.73 \| 13 \| 1 \| \|  \| A0A3S4XX79 \| Chaperonin GroEL OS=Mannheimia haemolytica OX=75985 GN=groL PE=3 SV=1 \| 29 \| 10 \| 20 \| 10 \| 546 \| 57.6 \| 5 \| 63.66 \| 10 \| 1 \| \|  \| A0A248ZXR5 \| Purine nucleoside phosphorylase DeoD-type OS=Mannheimia haemolytica OX=75985 GN=deoD PE=3 SV=1 \| 38 \| 6 \| 16 \| 6 \| 239 \| 26 \| 5.5 \| 56.76 \| 6 \| 1 \| \|  \| A0A3S4XDB1 \| Glutathione-binding protein gsiB OS=Mannheimia haemolytica OX=75985 GN=gsiB PE=3 SV=1 \| 23 \| 10 \| 19 \| 10 \| 527 \| 58 \| 7.31 \| 56.26 \| 10 \| 1 \| \|  \| A0A3S4XT40 \| Penicillin-binding protein activator LpoA OS=Mannheimia haemolytica OX=75985 GN=lpoA PE=4 SV=1 \| 8 \| 3 \| 17 \| 3 \| 572 \| 63.3 \| 5.36 \| 54.95 \| 3 \| 1 \| \|  \| A0A3S4XQ93 \| Pyruvate kinase OS=Mannheimia haemolytica OX=75985 GN=pykA PE=3 SV=1 \| 25 \| 7 \| 15 \| 7 \| 479 \| 51.6 \| 6.87 \| 47.1 \| 7 \| 1 \| \|  \| A0A249A2N2 \| Elongation factor Tu OS=Mannheimia haemolytica OX=75985 GN=tufA_2 PE=3 SV=1 \| 19 \| 6 \| 12 \| 6 \| 394 \| 43.3 \| 5.44 \| 43.3 \| 6 \| 1 \| \|  \| A0A3S5F331 \| Elongation factor G OS=Mannheimia haemolytica OX=75985 GN=fusA PE=3 SV=1 \| 22 \| 9 \| 13 \| 9 \| 700 \| 77 \| 5.2 \| 41.54 \| 9 \| 1 \| \|  \| A0A3S5BBG0 \| 2,3-bisphosphoglycerate-dependent phosphoglycerate mutase OS=Mannheimia haemolytica OX=75985 GN=gpmA PE=3 SV=1 \| 11 \| 2 \| 11 \| 2 \| 227 \| 26 \| 6.09 \| 39.97 \| 2 \| 1 \| \|  \| A0A448T9Y7 \| Cysteine synthase OS=Mannheimia haemolytica OX=75985 GN=cysK PE=3 SV=1 \| 26 \| 6 \| 13 \| 6 \| 315 \| 33.3 \| 6.62 \| 38.64 \| 6 \| 1 \| \|  \| A0A3S4XCM8 \| Aminotransferase OS=Mannheimia haemolytica OX=75985 GN=aspC PE=3 SV=1 \| 39 \| 9 \| 11 \| 9 \| 398 \| 43.4 \| 5.95 \| 37.06 \| 9 \| 1 \| \|  \| A0A3S4X9I0 \| Probable phosphomannomutase OS=Mannheimia haemolytica OX=75985 GN=NCTC10643_00522 PE=3 SV=1 \| 19 \| 7 \| 10 \| 7 \| 550 \| 59.8 \| 5.17 \| 35.97 \| 7 \| 1 \| \|  \| A0A448TCF5 \| Pyruvate dehydrogenase E1 component OS=Mannheimia haemolytica OX=75985 GN=aceE PE=4 SV=1 \| 15 \| 8 \| 9 \| 8 \| 885 \| 98.8 \| 5.68 \| 35.6 \| 8 \| 1 \| \|  \| A0A3S5B8E1 \| DNA-directed RNA polymerase subunit beta' OS=Mannheimia haemolytica OX=75985 GN=rpoC PE=3 SV=1 \| 8 \| 7 \| 10 \| 7 \| 1427 \| 157.9 \| 6.19 \| 34.14 \| 7 \| 1 \| \|  \| A0A448T7S1 \| Glutamine--fructose-6-phosphate aminotransferase [isomerizing] OS=Mannheimia haemolytica OX=75985 GN=glmS PE=3 SV=1 \| 18 \| 6 \| 8 \| 6 \| 611 \| 67.3 \| 5.29 \| 31.16 \| 6 \| 1 \| \|  \| A0A248ZXM7 \| Fructose-bisphosphate aldolase OS=Mannheimia haemolytica OX=75985 GN=fbaA PE=3 SV=1 \| 47 \| 10 \| 10 \| 10 \| 358 \| 39.1 \| 5.22 \| 31.12 \| 10 \| 1 \| \|  \| A0A3S4XZ36 \| Transferrin-binding protein 1 OS=Mannheimia haemolytica OX=75985 GN=tbp1 PE=3 SV=1 \| 11 \| 6 \| 9 \| 6 \| 936 \| 107.3 \| 8.94 \| 30.53 \| 6 \| 1 \| \|  \| A0A249A1S0 \| Enolase OS=Mannheimia haemolytica OX=75985 GN=eno PE=3 SV=1 \| 20 \| 5 \| 9 \| 5 \| 436 \| 46.1 \| 5.19 \| 29.02 \| 5 \| 1 \| \|  \| A0A3S4YI01 \| Phosphoenolpyruvate carboxykinase (ATP) OS=Mannheimia haemolytica OX=75985 GN=pckA PE=3 SV=1 \| 21 \| 9 \| 10 \| 9 \| 535 \| 59.4 \| 5.4 \| 28.88 \| 9 \| 1 \| \|  \| A0A448TA65 \| 5-methyltetrahydropteroyltriglutamate--homocysteine methyltransferase OS=Mannheimia haemolytica OX=75985 GN=metE_1 PE=4 SV=1 \| 16 \| 6 \| 9 \| 6 \| 380 \| 43.5 \| 5.31 \| 28 \| 6 \| 1 \| \|  \| Q51848 \| Putrescine-binding periplasmic protein OS=Mannheimia haemolytica OX=75985 GN=potD PE=3 SV=1 \| 23 \| 7 \| 10 \| 7 \| 364 \| 40.1 \| 5.12 \| 27.54 \| 7 \| 1 \| \|  \| A0A448TEW5 \| Malate dehydrogenase OS=Mannheimia haemolytica OX=75985 GN=mdh PE=3 SV=1 \| 22 \| 4 \| 8 \| 4 \| 320 \| 33.8 \| 5.52 \| 27.46 \| 4 \| 1 \| \|  \| A0A3S5B6R5 \| Phosphoglycerate kinase OS=Mannheimia haemolytica OX=75985 GN=pgk PE=3 SV=1 \| 20 \| 4 \| 8 \| 4 \| 391 \| 41.5 \| 5 \| 26.24 \| 4 \| 1 \| \|  \| A0A249A138 \| Dihydroxy-acid dehydratase OS=Mannheimia haemolytica OX=75985 GN=ilvD PE=3 SV=1 \| 16 \| 4 \| 7 \| 4 \| 615 \| 66.2 \| 5.78 \| 25.39 \| 4 \| 1 \| \|  \| A0A448TC90 \| Phosphoserine aminotransferase OS=Mannheimia haemolytica OX=75985 GN=serC PE=3 SV=1 \| 19 \| 4 \| 7 \| 4 \| 360 \| 39.9 \| 5.72 \| 25.1 \| 4 \| 1 \| \|  \| A0A249A0J9 \| Glyceraldehyde-3-phosphate dehydrogenase OS=Mannheimia haemolytica OX=75985 GN=gapA_2 PE=3 SV=1 \| 23 \| 3 \| 6 \| 3 \| 334 \| 35.7 \| 6.11 \| 24.87 \| 3 \| 1 \| \|  \| A0A448TA02 \| Transaldolase OS=Mannheimia haemolytica OX=75985 GN=talB PE=3 SV=1 \| 8 \| 2 \| 8 \| 2 \| 316 \| 35 \| 5.06 \| 24.8 \| 2 \| 1 \| \|  \| A0A3S4XLX6 \| Methylmalonate-semialdehyde dehydrogenase [acylating] OS=Mannheimia haemolytica OX=75985 GN=mmsA PE=4 SV=1 \| 24 \| 7 \| 8 \| 7 \| 503 \| 54 \| 5.66 \| 24.39 \| 7 \| 1 \| \|  \| A0A448T988 \| Isoleucine--tRNA ligase OS=Mannheimia haemolytica OX=75985 GN=ileS PE=3 SV=1 \| 12 \| 6 \| 6 \| 6 \| 938 \| 105.5 \| 5.71 \| 24 \| 6 \| 1 \| \|  \| A0A1D2Q7B1 \| Cysteine desulfurase IscS OS=Mannheimia haemolytica OX=75985 GN=iscS PE=3 SV=1 \| 16 \| 4 \| 7 \| 4 \| 406 \| 45.4 \| 6.11 \| 23.34 \| 4 \| 1 \| \|  \| A0A448TBI9 \| Polyribonucleotide nucleotidyltransferase OS=Mannheimia haemolytica OX=75985 GN=pnp PE=3 SV=1 \| 14 \| 6 \| 7 \| 6 \| 716 \| 77.6 \| 5.07 \| 23.26 \| 6 \| 1 \| \|  \| A0A248ZXQ9 \| 3-oxoacyl-[acyl-carrier-protein] synthase 1 OS=Mannheimia haemolytica OX=75985 GN=fabB PE=3 SV=1 \| 11 \| 2 \| 6 \| 2 \| 405 \| 42.6 \| 5.36 \| 23.14 \| 2 \| 1 \| \|  \| A0A3S4XDP7 \| Formate acetyltransferase OS=Mannheimia haemolytica OX=75985 GN=pflB PE=3 SV=1 \| 11 \| 5 \| 7 \| 5 \| 774 \| 86.7 \| 5.92 \| 22.8 \| 5 \| 1 \| \|  \| A0A249A192 \| Lipoprotein OS=Mannheimia haemolytica OX=75985 GN=metQ_3 PE=3 SV=1 \| 35 \| 4 \| 5 \| 4 \| 277 \| 30 \| 6.57 \| 22.52 \| 4 \| 1 \| \|  \| A0A448T6M1 \| Hemin-binding lipoprotein OS=Mannheimia haemolytica OX=75985 GN=hbpA_1 PE=3 SV=1 \| 22 \| 6 \| 7 \| 6 \| 529 \| 59.4 \| 6.81 \| 21.41 \| 6 \| 1 \| \|  \| A0A249A2P6 \| D-galactose/methyl-galactoside binding periplasmic protein MglB OS=Mannheimia haemolytica OX=75985 GN=mglB PE=3 SV=1 \| 26 \| 6 \| 6 \| 6 \| 329 \| 35.6 \| 5.85 \| 21.23 \| 6 \| 1 \| \|  \| A0A448TBF4 \| Phenylalanine--tRNA ligase beta subunit OS=Mannheimia haemolytica OX=75985 GN=pheT PE=3 SV=1 \| 9 \| 4 \| 5 \| 4 \| 795 \| 86.7 \| 5.19 \| 20.09 \| 4 \| 1 \| \|  \| A0A3S4YIS7 \| 2-hydroxypropyl-CoM lyase OS=Mannheimia haemolytica OX=75985 GN=xecA1 PE=4 SV=1 \| 8 \| 2 \| 4 \| 2 \| 343 \| 38.3 \| 5.33 \| 20.06 \| 2 \| 1 \| \|  \| A0A3S5BCS4 \| Cation efflux system protein CusC OS=Mannheimia haemolytica OX=75985 GN=cusC_2 PE=3 SV=1 \| 17 \| 4 \| 5 \| 4 \| 465 \| 51.4 \| 9.23 \| 19.48 \| 4 \| 1 \| \|  \| A0A378NBH1 \| Queuine tRNA-ribosyltransferase OS=Mannheimia haemolytica OX=75985 GN=tgt_4 PE=3 SV=1 \| 16 \| 3 \| 5 \| 3 \| 384 \| 43.9 \| 6.8 \| 18.51 \| 3 \| 1 \| \|  \| A0A3S4XP63 \| Acetate kinase OS=Mannheimia haemolytica OX=75985 GN=ackA PE=3 SV=1 \| 19 \| 4 \| 5 \| 4 \| 402 \| 43.5 \| 6.13 \| 18.31 \| 4 \| 1 \| \|  \| A0A248ZZM6 \| ATP-dependent 6-phosphofructokinase OS=Mannheimia haemolytica OX=75985 GN=pfkA PE=3 SV=1 \| 33 \| 4 \| 4 \| 4 \| 324 \| 35.4 \| 6.9 \| 17.73 \| 4 \| 1 \| \|  \| A0A3S5B6B8 \| Leukotoxin translocation ATP-binding protein LktB OS=Mannheimia haemolytica OX=75985 GN=gsiA_6 PE=4 SV=1 \| 16 \| 3 \| 4 \| 3 \| 268 \| 29.3 \| 8.88 \| 17.67 \| 3 \| 1 \| \|  \| A0A3S4WXU8 \| 2,3,4,5-tetrahydropyridine-2,6-dicarboxylate N-succinyltransferase OS=Mannheimia haemolytica OX=75985 GN=dapD PE=3 SV=1 \| 19 \| 4 \| 5 \| 4 \| 274 \| 29.4 \| 5.29 \| 17.67 \| 4 \| 1 \| \|  \| A0A248ZXM8 \| ATP synthase subunit alpha OS=Mannheimia haemolytica OX=75985 GN=atpA PE=3 SV=1 \| 10 \| 3 \| 5 \| 3 \| 513 \| 55.2 \| 5.72 \| 17.6 \| 3 \| 1 \| \|  \| A0A3S5BD43 \| Maltodextrin-binding protein OS=Mannheimia haemolytica OX=75985 GN=malE PE=3 SV=1 \| 18 \| 5 \| 6 \| 5 \| 396 \| 43.4 \| 6.3 \| 17.43 \| 5 \| 1 \| \|  \| A0A3S5F3L0 \| Acetyltransferase component of pyruvate dehydrogenase complex OS=Mannheimia haemolytica OX=75985 GN=aceF PE=3 SV=1 \| 12 \| 5 \| 6 \| 5 \| 634 \| 66.6 \| 5.2 \| 16.96 \| 5 \| 1 \| \|  \| A0A3S4YIG4 \| Periplasmic serine endoprotease DegP OS=Mannheimia haemolytica OX=75985 GN=degP PE=4 SV=1 \| 20 \| 6 \| 6 \| 6 \| 464 \| 49 \| 6.46 \| 16.46 \| 6 \| 1 \| \|  \| A0A448T3A2 \| Trigger factor OS=Mannheimia haemolytica OX=75985 GN=tig PE=3 SV=1 \| 12 \| 3 \| 5 \| 3 \| 432 \| 48.2 \| 5 \| 16.39 \| 3 \| 1 \| \|  \| A0A448T8W8 \| Threonine synthase OS=Mannheimia haemolytica OX=75985 GN=thrC PE=3 SV=1 \| 15 \| 4 \| 6 \| 4 \| 424 \| 46.4 \| 5.5 \| 16.34 \| 4 \| 1 \| \|  \| A0A3S4X0U9 \| Maltose-inducible porin OS=Mannheimia haemolytica OX=75985 GN=lamB PE=3 SV=1 \| 14 \| 5 \| 6 \| 5 \| 423 \| 47.3 \| 8.37 \| 16.04 \| 5 \| 1 \| \|  \| A0A448TEA5 \| Lipoprotein HlpB OS=Mannheimia haemolytica OX=75985 GN=NCTC10643_02184 PE=4 SV=1 \| 30 \| 4 \| 5 \| 4 \| 194 \| 20.7 \| 5.47 \| 15.92 \| 4 \| 1 \| \|  \| A0A448T9T6 \| N-acetylglucosamine-6-phosphate deacetylase OS=Mannheimia haemolytica OX=75985 GN=nagA PE=3 SV=1 \| 17 \| 3 \| 4 \| 3 \| 380 \| 41.1 \| 5.66 \| 15.91 \| 3 \| 1 \| \|  \| A0A3S4WUU9 \| 6-phosphogluconate dehydrogenase, decarboxylating OS=Mannheimia haemolytica OX=75985 GN=yqjI PE=3 SV=1 \| 10 \| 3 \| 4 \| 3 \| 484 \| 53.4 \| 5.38 \| 15.85 \| 3 \| 1 \| \|  \| A0A448T568 \| DNA-directed RNA polymerase subunit beta OS=Mannheimia haemolytica OX=75985 GN=rpoB PE=3 SV=1 \| 6 \| 5 \| 5 \| 5 \| 1341 \| 149.4 \| 5.25 \| 15.68 \| 5 \| 1 \| \|  \| A0A448TDZ6 \| Transferrin-binding protein B OS=Mannheimia haemolytica OX=75985 GN=tbpB PE=4 SV=1 \| 10 \| 4 \| 6 \| 4 \| 584 \| 63.4 \| 8.1 \| 15.24 \| 4 \| 1 \| \|  \| A0A3S4XDU0 \| ribonucleoside-diphosphate reductase OS=Mannheimia haemolytica OX=75985 GN=nrdB_2 PE=3 SV=1 \| 18 \| 4 \| 4 \| 4 \| 325 \| 38.4 \| 5.16 \| 15.08 \| 4 \| 1 \| \|  \| A0A3S4Z8A8 \| NAD(P) transhydrogenase subunit alpha OS=Mannheimia haemolytica OX=75985 GN=pntA PE=3 SV=1 \| 12 \| 3 \| 3 \| 3 \| 512 \| 54.6 \| 5.55 \| 14.73 \| 3 \| 1 \| \|  \| A0A448T3V8 \| Chaperone protein DnaK OS=Mannheimia haemolytica OX=75985 GN=dnaK PE=2 SV=1 \| 6 \| 2 \| 4 \| 2 \| 632 \| 68.2 \| 4.87 \| 14.63 \| 2 \| 1 \| \|  \| A0A448TBG6 \| 30S ribosomal protein S1 OS=Mannheimia haemolytica OX=75985 GN=rpsA PE=3 SV=1 \| 10 \| 3 \| 3 \| 3 \| 555 \| 60.1 \| 5.06 \| 14.43 \| 3 \| 1 \| \|  \| A0A448TDI0 \| UDP-glucose 4-epimerase OS=Mannheimia haemolytica OX=75985 GN=galE PE=3 SV=1 \| 18 \| 3 \| 4 \| 3 \| 338 \| 37.1 \| 6 \| 14.4 \| 3 \| 1 \| \|  \| A0A448TEX6 \| USG-1 protein OS=Mannheimia haemolytica OX=75985 GN=usg PE=4 SV=1 \| 25 \| 3 \| 4 \| 3 \| 328 \| 36 \| 4.55 \| 14.09 \| 3 \| 1 \| \|  \| A0A3S4XEH8 \| Iron uptake protein A1 OS=Mannheimia haemolytica OX=75985 GN=futA1 PE=4 SV=1 \| 10 \| 3 \| 5 \| 3 \| 342 \| 38 \| 7.84 \| 14.06 \| 3 \| 1 \| \|  \| A0A448TCB5 \| Dihydrolipoyl dehydrogenase OS=Mannheimia haemolytica OX=75985 GN=lpdA PE=3 SV=1 \| 15 \| 5 \| 5 \| 5 \| 474 \| 50.5 \| 6.07 \| 13.43 \| 5 \| 1 \| \|  \| A0A249A2L3 \| Small ribosomal subunit protein uS4 OS=Mannheimia haemolytica OX=75985 GN=rpsD PE=3 SV=1 \| 24 \| 3 \| 4 \| 3 \| 208 \| 23.8 \| 10.2 \| 13.35 \| 3 \| 1 \| \|  \| A0A3S4Z8G5 \| Outer membrane protein P4 OS=Mannheimia haemolytica OX=75985 GN=hel PE=4 SV=1 \| 21 \| 4 \| 5 \| 4 \| 273 \| 30.8 \| 8.97 \| 13.21 \| 4 \| 1 \| \|  \| A0A378N2S0 \| Outer membrane protein assembly factor BamD OS=Mannheimia haemolytica OX=75985 GN=bamD PE=3 SV=1 \| 18 \| 3 \| 4 \| 3 \| 259 \| 29.7 \| 5.8 \| 12.98 \| 3 \| 1 \| \|  \| A0A3S4YGK0 \| Chaperone protein ClpB OS=Mannheimia haemolytica OX=75985 GN=clpB PE=3 SV=1 \| 8 \| 4 \| 4 \| 4 \| 855 \| 95.5 \| 5.66 \| 12.82 \| 4 \| 1 \| \|  \| A0A448T8S8 \| Protease 3 OS=Mannheimia haemolytica OX=75985 GN=ptrA PE=3 SV=1 \| 6 \| 3 \| 3 \| 3 \| 981 \| 110.1 \| 5.94 \| 12.38 \| 3 \| 1 \| \|  \| A0A448TC92 \| Asparagine--tRNA ligase OS=Mannheimia haemolytica OX=75985 GN=asnS PE=3 SV=1 \| 12 \| 3 \| 3 \| 3 \| 467 \| 52.8 \| 5.31 \| 12.37 \| 3 \| 1 \| \|  \| A0A448T6H4 \| Glycerophosphoryl diester phosphodiesterase OS=Mannheimia haemolytica OX=75985 GN=glpQ PE=4 SV=1 \| 14 \| 3 \| 3 \| 3 \| 357 \| 41.1 \| 6.55 \| 12.06 \| 3 \| 1 \| \|  \| A0A3S5BCF9 \| EIIBC-Fru OS=Mannheimia haemolytica OX=75985 GN=fruA PE=4 SV=1 \| 11 \| 2 \| 2 \| 2 \| 551 \| 56.8 \| 7.11 \| 11.46 \| 2 \| 1 \| \|  \| A0A3S4XQ45 \| Aminopeptidase N OS=Mannheimia haemolytica OX=75985 GN=pepN PE=3 SV=1 \| 4 \| 2 \| 3 \| 2 \| 870 \| 99.9 \| 5.36 \| 11.18 \| 2 \| 1 \| \|  \| A0A448TDV3 \| Glutathione reductase OS=Mannheimia haemolytica OX=75985 GN=gor PE=3 SV=1 \| 13 \| 3 \| 3 \| 3 \| 456 \| 49 \| 6.1 \| 11.17 \| 3 \| 1 \| \|  \| A0A248ZXK7 \| N-acetylneuraminate lyase OS=Mannheimia haemolytica OX=75985 GN=nanA PE=3 SV=1 \| 19 \| 4 \| 4 \| 4 \| 292 \| 32.6 \| 5.6 \| 11.16 \| 4 \| 1 \| \|  \| A0A3S5BD34 \| PTS-dependent dihydroxyacetone kinase, dihydroxyacetone-binding subunit dhaK OS=Mannheimia haemolytica OX=75985 GN=dhaK PE=4 SV=1 \| 14 \| 3 \| 3 \| 3 \| 356 \| 38.5 \| 5.12 \| 11.14 \| 3 \| 1 \| \|  \| A0A448T8E1 \| Inositol 2-dehydrogenase OS=Mannheimia haemolytica OX=75985 GN=idhA PE=4 SV=1 \| 7 \| 1 \| 3 \| 1 \| 336 \| 36.5 \| 5.74 \| 11.12 \| 1 \| 1 \| \|  \| A0A448TAG2 \| Protein translocase subunit SecA OS=Mannheimia haemolytica OX=75985 GN=secA PE=3 SV=1 \| 5 \| 3 \| 3 \| 3 \| 908 \| 102.3 \| 5.45 \| 11.05 \| 3 \| 1 \| \|  \| A0A3S5B6Y6 \| Serine hydroxymethyltransferase OS=Mannheimia haemolytica OX=75985 GN=glyA PE=3 SV=1 \| 9 \| 2 \| 3 \| 2 \| 420 \| 45.6 \| 6.67 \| 10.92 \| 2 \| 1 \| \|  \| A0A248ZW42 \| ATP synthase subunit beta OS=Mannheimia haemolytica OX=75985 GN=atpD PE=3 SV=1 \| 13 \| 3 \| 3 \| 3 \| 457 \| 49.8 \| 5.17 \| 10.9 \| 3 \| 1 \| \|  \| A0A448TD54 \| Bifunctional protein HldE OS=Mannheimia haemolytica OX=75985 GN=hldE PE=3 SV=1 \| 8 \| 2 \| 3 \| 2 \| 475 \| 51.5 \| 5.63 \| 10.62 \| 2 \| 1 \| \|  \| A0A249A2K7 \| D-ribose-binding periplasmic protein OS=Mannheimia haemolytica OX=75985 GN=rbsB_1 PE=4 SV=1 \| 16 \| 3 \| 4 \| 3 \| 312 \| 33.6 \| 6.35 \| 10.49 \| 3 \| 1 \| \|  \| A0A3S5B1Q3 \| Outer membrane protein OS=Mannheimia haemolytica OX=75985 GN=NCTC10643_00367 PE=4 SV=1 \| 10 \| 3 \| 4 \| 3 \| 314 \| 37.1 \| 9.17 \| 10.49 \| 3 \| 1 \| \|  \| A0A448TBM6 \| GMP synthase [glutamine-hydrolyzing] OS=Mannheimia haemolytica OX=75985 GN=guaA PE=3 SV=1 \| 8 \| 2 \| 2 \| 2 \| 523 \| 58.1 \| 6.23 \| 10.31 \| 2 \| 1 \| \|  \| A0A448T9M9 \| Glucose-6-phosphate isomerase OS=Mannheimia haemolytica OX=75985 GN=pgi PE=3 SV=1 \| 10 \| 4 \| 4 \| 4 \| 546 \| 61.1 \| 6.68 \| 10.23 \| 4 \| 1 \| \|  \| A0A3S4YGX2 \| Elongation factor Ts OS=Mannheimia haemolytica OX=75985 GN=tsf PE=3 SV=1 \| 14 \| 3 \| 4 \| 3 \| 283 \| 30.1 \| 5.05 \| 10.13 \| 3 \| 1 \| \|  \| A0A3S4XLQ0 \| Glycine--tRNA ligase beta subunit OS=Mannheimia haemolytica OX=75985 GN=glyS PE=3 SV=1 \| 6 \| 2 \| 2 \| 2 \| 688 \| 75.6 \| 5.34 \| 10.05 \| 2 \| 1 \| \|  \| A0A3S4Z398 \| Probable amino-acid ABC transporter-binding protein HI_1080 OS=Mannheimia haemolytica OX=75985 GN=NCTC10643_00175 PE=3 SV=1 \| 16 \| 3 \| 3 \| 3 \| 257 \| 28 \| 6.64 \| 10.04 \| 3 \| 1 \| \|  \| A0A248ZWV5 \| Catalase OS=Mannheimia haemolytica OX=75985 GN=katA PE=3 SV=1 \| 9 \| 3 \| 4 \| 3 \| 502 \| 56.9 \| 6.74 \| 9.92 \| 3 \| 1 \| \|  \| A0A448T5J9 \| Pyridoxal kinase PdxY OS=Mannheimia haemolytica OX=75985 GN=pdxY PE=3 SV=1 \| 18 \| 3 \| 3 \| 3 \| 286 \| 31.2 \| 6.44 \| 9.89 \| 3 \| 1 \| \|  \| A0A448TE00 \| Thioredoxin reductase OS=Mannheimia haemolytica OX=75985 GN=trxB PE=3 SV=1 \| 15 \| 2 \| 2 \| 2 \| 317 \| 34.3 \| 5.52 \| 9.88 \| 2 \| 1 \| \|  \| A0A448TAZ5 \| NADP-dependent malic enzyme OS=Mannheimia haemolytica OX=75985 GN=maeB PE=3 SV=1 \| 11 \| 4 \| 4 \| 4 \| 441 \| 47.4 \| 5.21 \| 9.61 \| 4 \| 1 \| \|  \| A0A448TAV7 \| Leukotoxin translocation ATP-binding protein LktB OS=Mannheimia haemolytica OX=75985 GN=NCTC10643_01136 PE=4 SV=1 \| 3 \| 1 \| 2 \| 1 \| 643 \| 72.8 \| 6.42 \| 9.57 \| 1 \| 1 \| \|  \| A0A1D2Q5P1 \| 3-oxoacyl-[acyl-carrier-protein] reductase OS=Mannheimia haemolytica OX=75985 GN=fabG PE=3 SV=1 \| 15 \| 2 \| 3 \| 2 \| 240 \| 25.2 \| 5.73 \| 9.46 \| 2 \| 1 \| \|  \| A0A3S5F347 \| Chaperone protein DnaJ OS=Mannheimia haemolytica OX=75985 GN=dnaJ PE=3 SV=1 \| 8 \| 2 \| 3 \| 2 \| 370 \| 39.8 \| 7.43 \| 9.39 \| 2 \| 1 \| \|  \| A0A448TAC4 \| Tol-Pal system protein TolB OS=Mannheimia haemolytica OX=75985 GN=tolB PE=3 SV=1 \| 8 \| 2 \| 3 \| 2 \| 428 \| 44.7 \| 8.19 \| 9.39 \| 2 \| 1 \| \|  \| A0A448T303 \| Glucose-6-phosphate 1-dehydrogenase OS=Mannheimia haemolytica OX=75985 GN=zwf PE=3 SV=1 \| 4 \| 1 \| 2 \| 1 \| 495 \| 56.1 \| 6.76 \| 9.03 \| 1 \| 1 \| \|  \| A0A448T2I6 \| Uroporphyrinogen decarboxylase OS=Mannheimia haemolytica OX=75985 GN=hemE PE=3 SV=1 \| 10 \| 2 \| 2 \| 2 \| 354 \| 39.2 \| 6.7 \| 8.99 \| 2 \| 1 \| \|  \| A0A3S4WZ88 \| Uridylate kinase OS=Mannheimia haemolytica OX=75985 GN=pyrH PE=3 SV=1 \| 13 \| 2 \| 2 \| 2 \| 238 \| 25.8 \| 6.37 \| 8.94 \| 2 \| 1 \| \|  \| A0A3S4WUP7 \| Ribonuclease E OS=Mannheimia haemolytica OX=75985 GN=rne PE=3 SV=1 \| 4 \| 2 \| 2 \| 2 \| 980 \| 111.4 \| 7.28 \| 8.83 \| 2 \| 1 \| \|  \| A0A3S5BAD1 \| Diaminobutyrate--2-oxoglutarate aminotransferase OS=Mannheimia haemolytica OX=75985 GN=dat PE=3 SV=1 \| 7 \| 1 \| 2 \| 1 \| 454 \| 49.2 \| 7.49 \| 8.83 \| 1 \| 1 \| \|  \| A0A3S4YH40 \| Outer membrane protein P5 OS=Mannheimia haemolytica OX=75985 GN=ompA_2 PE=4 SV=1 \| 14 \| 3 \| 3 \| 3 \| 284 \| 31.4 \| 8.46 \| 8.7 \| 3 \| 1 \| \|  \| A0A448TEL0 \| Inositol-1-monophosphatase OS=Mannheimia haemolytica OX=75985 GN=suhB PE=3 SV=1 \| 10 \| 2 \| 3 \| 2 \| 270 \| 29.6 \| 6.58 \| 8.64 \| 2 \| 1 \| \|  \| A0A448TBT6 \| Cystathionine gamma-synthase/O-acetylhomoserine (Thiol)-lyase OS=Mannheimia haemolytica OX=75985 GN=metI_1 PE=3 SV=1 \| 12 \| 3 \| 3 \| 3 \| 377 \| 41.3 \| 6.3 \| 8.47 \| 3 \| 1 \| \|  \| A0A3S4XFU5 \| Probable endonuclease 4 OS=Mannheimia haemolytica OX=75985 GN=nfo PE=3 SV=1 \| 14 \| 2 \| 2 \| 2 \| 281 \| 31.5 \| 6.47 \| 8.28 \| 2 \| 1 \| \|  \| A0A3S4XDV3 \| Superoxide dismutase OS=Mannheimia haemolytica OX=75985 GN=sodA PE=3 SV=1 \| 18 \| 3 \| 3 \| 3 \| 213 \| 23.9 \| 6.27 \| 8.04 \| 3 \| 1 \| \|  \| A0A3S4XE23 \| Porphobilinogen deaminase OS=Mannheimia haemolytica OX=75985 GN=hemC PE=3 SV=1 \| 14 \| 2 \| 2 \| 2 \| 311 \| 33.7 \| 6.25 \| 8.04 \| 2 \| 1 \| \|  \| A0A448T6N3 \| 2,3-diketo-L-gulonate reductase OS=Mannheimia haemolytica OX=75985 GN=dlgD PE=3 SV=1 \| 7 \| 1 \| 2 \| 1 \| 334 \| 36.9 \| 5.36 \| 7.98 \| 1 \| 1 \| \|  \| A0A3S4X0X9 \| Threonine--tRNA ligase OS=Mannheimia haemolytica OX=75985 GN=thrS PE=3 SV=1 \| 4 \| 1 \| 2 \| 1 \| 643 \| 73.6 \| 6 \| 7.9 \| 1 \| 1 \| \|  \| A0A3S5B677 \| Aspartate-semialdehyde dehydrogenase OS=Mannheimia haemolytica OX=75985 GN=asd PE=3 SV=1 \| 11 \| 2 \| 2 \| 2 \| 371 \| 40.5 \| 5.31 \| 7.89 \| 2 \| 1 \| \|  \| A0A448TCG7 \| Bifunctional protein GlmU OS=Mannheimia haemolytica OX=75985 GN=glmU PE=3 SV=1 \| 4 \| 1 \| 2 \| 1 \| 454 \| 49 \| 6.7 \| 7.88 \| 1 \| 1 \| \|  \| A0A3S4XMF2 \| Ketol-acid reductoisomerase (NADP(+)) OS=Mannheimia haemolytica OX=75985 GN=ilvC PE=3 SV=1 \| 6 \| 2 \| 2 \| 2 \| 493 \| 54.2 \| 5.01 \| 7.79 \| 2 \| 1 \| \|  \| A0A448TB87 \| Enoyl-[acyl-carrier-protein] reductase [NADH] OS=Mannheimia haemolytica OX=75985 GN=fabI PE=3 SV=1 \| 19 \| 3 \| 3 \| 3 \| 263 \| 28.2 \| 5.24 \| 7.69 \| 3 \| 1 \| \|  \| A0A3S4WZT8 \| Phosphate acetyltransferase OS=Mannheimia haemolytica OX=75985 GN=pta PE=3 SV=1 \| 7 \| 2 \| 2 \| 2 \| 711 \| 76.5 \| 5.71 \| 7.68 \| 2 \| 1 \| \|  \| A0A448TCN5 \| Outer membrane protein assembly factor BamA OS=Mannheimia haemolytica OX=75985 GN=yaeT PE=3 SV=1 \| 5 \| 3 \| 3 \| 3 \| 793 \| 88.8 \| 6.23 \| 7.58 \| 3 \| 1 \| \|  \| A0A448TD08 \| Transketolase OS=Mannheimia haemolytica OX=75985 GN=tktA PE=3 SV=1 \| 6 \| 3 \| 3 \| 3 \| 668 \| 73.5 \| 5.72 \| 7.55 \| 3 \| 1 \| \|  \| A0A249A2W3 \| DNA-directed RNA polymerase subunit alpha OS=Mannheimia haemolytica OX=75985 GN=rpoA PE=3 SV=1 \| 9 \| 2 \| 2 \| 2 \| 329 \| 36.4 \| 5.33 \| 7.47 \| 2 \| 1 \| \|  \| A0A3S4XIQ0 \| Ribonuclease R OS=Mannheimia haemolytica OX=75985 GN=rnr PE=3 SV=1 \| 3 \| 1 \| 2 \| 1 \| 787 \| 90.1 \| 7.08 \| 7.44 \| 1 \| 1 \| \|  \| A0A1D2Q7D5 \| Protein RecA OS=Mannheimia haemolytica OX=75985 GN=recA PE=3 SV=1 \| 8 \| 2 \| 2 \| 2 \| 368 \| 39.6 \| 5.2 \| 7.28 \| 2 \| 1 \| \|  \| A0A448TBL6 \| Multidrug resistance protein mexA OS=Mannheimia haemolytica OX=75985 GN=mexA PE=3 SV=1 \| 8 \| 2 \| 2 \| 2 \| 401 \| 43.2 \| 9.22 \| 7.19 \| 2 \| 1 \| \|  \| A0A448TA83 \| Adenylosuccinate synthetase OS=Mannheimia haemolytica OX=75985 GN=purA PE=3 SV=1 \| 8 \| 2 \| 2 \| 2 \| 432 \| 47.5 \| 5.8 \| 7.16 \| 2 \| 1 \| \|  \| A0A3S4WZ37 \| Phosphoenolpyruvate carboxylase OS=Mannheimia haemolytica OX=75985 GN=ppc PE=3 SV=1 \| 6 \| 2 \| 2 \| 2 \| 879 \| 99.6 \| 6.13 \| 7.1 \| 2 \| 1 \| \|  \| A0A448T9X1 \| Probable cytosol aminopeptidase OS=Mannheimia haemolytica OX=75985 GN=pepA PE=3 SV=1 \| 6 \| 2 \| 2 \| 2 \| 498 \| 54.1 \| 6.62 \| 7.02 \| 2 \| 1 \| \|  \| A0A3S4XNZ9 \| Aspartokinase OS=Mannheimia haemolytica OX=75985 GN=lysC PE=3 SV=1 \| 9 \| 2 \| 3 \| 2 \| 450 \| 48.3 \| 5.57 \| 6.98 \| 2 \| 1 \| \|  \| A0A448TDW2 \| UDP-N-acetylglucosamine 1-carboxyvinyltransferase OS=Mannheimia haemolytica OX=75985 GN=murA PE=3 SV=1 \| 8 \| 2 \| 2 \| 2 \| 426 \| 45.9 \| 6.06 \| 6.97 \| 2 \| 1 \| \|  \| A0A3S5F3D7 \| Hemin-binding lipoprotein OS=Mannheimia haemolytica OX=75985 GN=hbpA_2 PE=3 SV=1 \| 6 \| 2 \| 2 \| 2 \| 547 \| 60.9 \| 6.48 \| 6.93 \| 2 \| 1 \| \|  \| A0A3S4X9H0 \| Chaperone protein HtpG OS=Mannheimia haemolytica OX=75985 GN=htpG PE=3 SV=1 \| 5 \| 2 \| 2 \| 2 \| 627 \| 71.1 \| 5.16 \| 6.87 \| 2 \| 1 \| \|  \| A0A3S4XII9 \| Cys regulon transcriptional activator OS=Mannheimia haemolytica OX=75985 GN=cysB PE=3 SV=1 \| 15 \| 2 \| 2 \| 2 \| 327 \| 37 \| 6.65 \| 6.81 \| 2 \| 1 \| \|  \| A0A3S4XDL5 \| Cytosol non-specific dipeptidase OS=Mannheimia haemolytica OX=75985 GN=pepD PE=4 SV=1 \| 5 \| 1 \| 1 \| 1 \| 484 \| 52.9 \| 5.14 \| 6.8 \| 1 \| 1 \| \|  \| A0A448T9J2 \| Tyrosine--tRNA ligase OS=Mannheimia haemolytica OX=75985 GN=tyrS PE=3 SV=1 \| 9 \| 2 \| 2 \| 2 \| 396 \| 44.3 \| 5.83 \| 6.71 \| 2 \| 1 \| \|  \| A0A448TAH2 \| LPS-assembly protein LptD OS=Mannheimia haemolytica OX=75985 GN=lptD PE=3 SV=1 \| 6 \| 2 \| 2 \| 2 \| 777 \| 89.3 \| 8.25 \| 6.62 \| 2 \| 1 \| \|  \| A0A448T3Y1 \| Gamma-glutamyl phosphate reductase OS=Mannheimia haemolytica OX=75985 GN=proA PE=3 SV=1 \| 9 \| 2 \| 2 \| 2 \| 412 \| 45.1 \| 5.41 \| 6.56 \| 2 \| 1 \| \|  \| A0A3S4X783 \| Osmotically-inducible protein Y OS=Mannheimia haemolytica OX=75985 GN=osmY PE=4 SV=1 \| 17 \| 2 \| 2 \| 2 \| 196 \| 20.9 \| 5.72 \| 6.56 \| 2 \| 1 \| \|  \| A0A448T241 \| Beta sliding clamp OS=Mannheimia haemolytica OX=75985 GN=dnaN PE=3 SV=1 \| 7 \| 1 \| 1 \| 1 \| 367 \| 41.1 \| 5.12 \| 6.56 \| 1 \| 1 \| \|  \| A0A448T5M4 \| Fumarate hydratase class II OS=Mannheimia haemolytica OX=75985 GN=fumC PE=3 SV=1 \| 9 \| 2 \| 2 \| 2 \| 464 \| 50.4 \| 6.48 \| 6.43 \| 2 \| 1 \| \|  \| A0A448TDD5 \| 4-hydroxy-tetrahydrodipicolinate synthase OS=Mannheimia haemolytica OX=75985 GN=dapA PE=3 SV=1 \| 9 \| 2 \| 2 \| 2 \| 295 \| 31.5 \| 5.62 \| 6.39 \| 2 \| 1 \| \|  \| A0A3S4X974 \| Fumarate reductase flavoprotein subunit OS=Mannheimia haemolytica OX=75985 GN=frdA PE=3 SV=1 \| 5 \| 2 \| 2 \| 2 \| 600 \| 66.1 \| 6.3 \| 6.23 \| 2 \| 1 \| \|  \| A0A448TCW7 \| Multifunctional fusion protein OS=Mannheimia haemolytica OX=75985 GN=trpC PE=3 SV=1 \| 4 \| 1 \| 2 \| 1 \| 478 \| 52.9 \| 5.94 \| 6.18 \| 1 \| 1 \| \|  \| A0A448TCY6 \| Protein QmcA OS=Mannheimia haemolytica OX=75985 GN=qmcA PE=3 SV=1 \| 11 \| 2 \| 2 \| 2 \| 306 \| 33.9 \| 5.64 \| 6.12 \| 2 \| 1 \| \|  \| A0A448TBM5 \| Hemolysin OS=Mannheimia haemolytica OX=75985 GN=hpmA PE=3 SV=1 \| 2 \| 2 \| 2 \| 2 \| 2983 \| 322.1 \| 6.54 \| 6.11 \| 2 \| 1 \| \|  \| A0A3S4XGI3 \| Aspartate ammonia-lyase OS=Mannheimia haemolytica OX=75985 GN=aspA PE=3 SV=1 \| 7 \| 2 \| 2 \| 2 \| 475 \| 51.7 \| 5.07 \| 6.06 \| 2 \| 1 \| \|  \| A0A3S5B8L0 \| Membrane protein insertase YidC OS=Mannheimia haemolytica OX=75985 GN=yidC PE=3 SV=1 \| 5 \| 2 \| 2 \| 2 \| 541 \| 60.5 \| 8.29 \| 6.02 \| 2 \| 1 \| \|  \| A0A448T710 \| Mannitol-1-phosphate 5-dehydrogenase OS=Mannheimia haemolytica OX=75985 GN=mtlD PE=3 SV=1 \| 7 \| 1 \| 1 \| 1 \| 379 \| 41.9 \| 5.17 \| 5.99 \| 1 \| 1 \| \|  \| A0A3S4YGH8 \| Uroporphyrinogen-III C-methyltransferase OS=Mannheimia haemolytica OX=75985 GN=hemX PE=4 SV=1 \| 9 \| 2 \| 2 \| 2 \| 436 \| 48.7 \| 5.06 \| 5.91 \| 2 \| 1 \| \|  \| A0A249A152 \| Aminopeptidase PepB OS=Mannheimia haemolytica OX=75985 GN=pepB PE=3 SV=1 \| 7 \| 1 \| 1 \| 1 \| 426 \| 46.2 \| 5.86 \| 5.86 \| 1 \| 1 \| \|  \| A0A448TBW4 \| EIICBA-Glc OS=Mannheimia haemolytica OX=75985 GN=ptsG_2 PE=4 SV=1 \| 4 \| 1 \| 1 \| 1 \| 483 \| 51.4 \| 8.38 \| 5.78 \| 1 \| 1 \| \|  \| A0A448TDY0 \| Branched-chain-amino-acid aminotransferase OS=Mannheimia haemolytica OX=75985 GN=ilvE PE=3 SV=1 \| 4 \| 1 \| 2 \| 1 \| 345 \| 38.1 \| 5.85 \| 5.75 \| 1 \| 1 \| \|  \| A0A3S5F3D1 \| Biotin carboxylase OS=Mannheimia haemolytica OX=75985 GN=accC PE=4 SV=1 \| 4 \| 1 \| 2 \| 1 \| 447 \| 49.2 \| 7.21 \| 5.74 \| 1 \| 1 \| \|  \| A0A448T693 \| Proline--tRNA ligase OS=Mannheimia haemolytica OX=75985 GN=proS PE=3 SV=1 \| 4 \| 2 \| 2 \| 2 \| 571 \| 63.5 \| 5.29 \| 5.64 \| 2 \| 1 \| \|  \| A0A3S5BCR8 \| Lipoprotein OS=Mannheimia haemolytica OX=75985 GN=metQ_2 PE=3 SV=1 \| 8 \| 1 \| 1 \| 1 \| 275 \| 30.1 \| 6.14 \| 5.61 \| 1 \| 1 \| \|  \| A0A448T8M7 \| L-2,4-diaminobutyrate decarboxylase OS=Mannheimia haemolytica OX=75985 GN=ddc PE=3 SV=1 \| 5 \| 1 \| 1 \| 1 \| 511 \| 56 \| 5.74 \| 5.54 \| 1 \| 1 \| \|  \| A0A448TDL1 \| Aspartate--tRNA ligase OS=Mannheimia haemolytica OX=75985 GN=aspS PE=3 SV=1 \| 2 \| 1 \| 2 \| 1 \| 588 \| 66.4 \| 5.52 \| 5.49 \| 1 \| 1 \| \|  \| A0A378N2A5 \| PTS system mannose-specific EIIAB component OS=Mannheimia haemolytica OX=75985 GN=manX_1 PE=4 SV=1 \| 7 \| 1 \| 1 \| 1 \| 325 \| 35.3 \| 5 \| 5.42 \| 1 \| 1 \| \|  \| A0A448T9A5 \| 23S rRNA (guanosine-2'-O-)-methyltransferase RlmB OS=Mannheimia haemolytica OX=75985 GN=rlmB PE=3 SV=1 \| 8 \| 1 \| 1 \| 1 \| 246 \| 26.7 \| 7.09 \| 5.4 \| 1 \| 1 \| \|  \| A0A3S4XBM2 \| GTP cyclohydrolase 1 OS=Mannheimia haemolytica OX=75985 GN=folE PE=3 SV=1 \| 10 \| 1 \| 1 \| 1 \| 229 \| 25.9 \| 6.3 \| 5.39 \| 1 \| 1 \| \|  \| A0A448T548 \| Glutamine synthetase OS=Mannheimia haemolytica OX=75985 GN=glnA PE=3 SV=1 \| 4 \| 1 \| 1 \| 1 \| 473 \| 52.4 \| 5.34 \| 5.35 \| 1 \| 1 \| \|  \| A0A3S4XA81 \| Membrane-bound lytic murein transglycosylase C OS=Mannheimia haemolytica OX=75985 GN=emtA PE=3 SV=1 \| 6 \| 1 \| 1 \| 1 \| 363 \| 40.7 \| 9.47 \| 5.19 \| 1 \| 1 \| \|  \| A0A3S5B5Q3 \| 2',3'-cyclic-nucleotide 2'-phosphodiesterase/3'-nucleotidase OS=Mannheimia haemolytica OX=75985 GN=cpdB PE=3 SV=1 \| 4 \| 2 \| 2 \| 2 \| 658 \| 73.1 \| 6.27 \| 5.18 \| 2 \| 1 \| \|  \| A0A448TA64 \| Putative protoheme IX biogenesis protein OS=Mannheimia haemolytica OX=75985 GN=hemY PE=4 SV=1 \| 5 \| 1 \| 1 \| 1 \| 421 \| 47.8 \| 6 \| 5.11 \| 1 \| 1 \| \|  \| A0A3S4XQE5 \| Domain of uncharacterized function (DUF1852) OS=Mannheimia haemolytica OX=75985 GN=NCTC10643_02214 PE=4 SV=1 \| 8 \| 1 \| 1 \| 1 \| 325 \| 37.6 \| 6.34 \| 5.11 \| 1 \| 1 \| \|  \| A0A3S5B0U7 \| Bifunctional phosphatase/peptidyl-prolyl cis-trans isomerase OS=Mannheimia haemolytica OX=75985 GN=NCTC10643_00201 PE=4 SV=1 \| 5 \| 1 \| 2 \| 1 \| 270 \| 30.3 \| 5.26 \| 5.07 \| 1 \| 1 \| \|  \| A0A3S5BCM1 \| Cystathionine beta-lyase metC OS=Mannheimia haemolytica OX=75985 GN=metC PE=3 SV=1 \| 5 \| 1 \| 1 \| 1 \| 396 \| 44.1 \| 6.84 \| 5.06 \| 1 \| 1 \| \|  \| A0A3S4YFZ0 \| Putative glucose-6-phosphate 1-epimerase OS=Mannheimia haemolytica OX=75985 GN=yeaD PE=3 SV=1 \| 11 \| 2 \| 2 \| 2 \| 263 \| 29.6 \| 6.15 \| 5.04 \| 2 \| 1 \| \|  \| A0A3S4XDR8 \| Thiamine-phosphate synthase OS=Mannheimia haemolytica OX=75985 GN=thiE PE=3 SV=1 \| 4 \| 1 \| 1 \| 1 \| 509 \| 55.5 \| 6.09 \| 4.96 \| 1 \| 1 \| \|  \| A0A448T8W7 \| ATP-dependent RNA helicase SrmB OS=Mannheimia haemolytica OX=75985 GN=srmB PE=3 SV=1 \| 5 \| 1 \| 1 \| 1 \| 444 \| 50.2 \| 9.63 \| 4.93 \| 1 \| 1 \| \|  \| A0A248ZZM9 \| MurR/RpiR family transcriptional regulator OS=Mannheimia haemolytica OX=75985 GN=ybbH_2 PE=4 SV=1 \| 12 \| 2 \| 2 \| 2 \| 289 \| 32.1 \| 7.49 \| 4.88 \| 2 \| 1 \| \|  \| A0A3S4XEU0 \| Alanine--tRNA ligase OS=Mannheimia haemolytica OX=75985 GN=alaS PE=3 SV=1 \| 4 \| 2 \| 2 \| 2 \| 875 \| 96.3 \| 5.63 \| 4.88 \| 2 \| 1 \| \|  \| A0A448T2D5 \| Tail-specific protease OS=Mannheimia haemolytica OX=75985 GN=prc PE=3 SV=1 \| 4 \| 2 \| 2 \| 2 \| 670 \| 75.9 \| 8.24 \| 4.84 \| 2 \| 1 \| \|  \| A0A3S5F3J8 \| Citrate synthase OS=Mannheimia haemolytica OX=75985 GN=gltA PE=3 SV=1 \| 4 \| 1 \| 1 \| 1 \| 426 \| 48.2 \| 6.83 \| 4.84 \| 1 \| 1 \| \|  \| A0A3S4X9U9 \| Aspartate--ammonia ligase OS=Mannheimia haemolytica OX=75985 GN=asnA PE=3 SV=1 \| 4 \| 1 \| 2 \| 1 \| 330 \| 37.5 \| 5.52 \| 4.76 \| 1 \| 1 \| \|  \| A0A3S4Z7G6 \| Bor protein OS=Mannheimia haemolytica OX=75985 GN=NCTC10643_01275 PE=4 SV=1 \| 27 \| 1 \| 1 \| 1 \| 98 \| 10.4 \| 8.29 \| 4.71 \| 1 \| 1 \| \|  \| A0A3S4XQ75 \| 3-dehydroquinate synthase OS=Mannheimia haemolytica OX=75985 GN=aroB PE=3 SV=1 \| 7 \| 1 \| 1 \| 1 \| 362 \| 39.7 \| 5.97 \| 4.7 \| 1 \| 1 \| \|  \| A0A1D2Q776 \| Leukotoxin translocation ATP-binding protein LktB OS=Mannheimia haemolytica OX=75985 GN=artP PE=3 SV=1 \| 8 \| 1 \| 1 \| 1 \| 244 \| 27.1 \| 6.43 \| 4.67 \| 1 \| 1 \| \|  \| A0A3S4YHC0 \| Transcription termination/antitermination protein NusA OS=Mannheimia haemolytica OX=75985 GN=nusA PE=3 SV=1 \| 3 \| 1 \| 1 \| 1 \| 498 \| 55.4 \| 4.7 \| 4.67 \| 1 \| 1 \| \|  \| A0A3S5B2W5 \| ATP synthase gamma chain OS=Mannheimia haemolytica OX=75985 GN=atpG PE=3 SV=1 \| 7 \| 1 \| 1 \| 1 \| 288 \| 31.8 \| 8.72 \| 4.65 \| 1 \| 1 \| \|  \| A0A448T815 \| 5-dehydro-2-deoxygluconokinase OS=Mannheimia haemolytica OX=75985 GN=iolC_1 PE=4 SV=1 \| 5 \| 1 \| 1 \| 1 \| 638 \| 70.9 \| 5.77 \| 4.64 \| 1 \| 1 \| \|  \| A0A3S4XZ46 \| 3-deoxy-manno-octulosonate cytidylyltransferase OS=Mannheimia haemolytica OX=75985 GN=kdsB PE=3 SV=1 \| 11 \| 1 \| 1 \| 1 \| 251 \| 28 \| 6.07 \| 4.55 \| 1 \| 1 \| \|  \| A0A448TCE9 \| Nitronate monooxygenase OS=Mannheimia haemolytica OX=75985 GN=NCTC10643_01476 PE=4 SV=1 \| 5 \| 1 \| 2 \| 1 \| 315 \| 34.2 \| 5.22 \| 4.51 \| 1 \| 1 \| \|  \| A0A3S4XDF7 \| Fructose-1,6-bisphosphatase class 1 OS=Mannheimia haemolytica OX=75985 GN=fbp PE=3 SV=1 \| 6 \| 1 \| 1 \| 1 \| 334 \| 36.9 \| 6 \| 4.44 \| 1 \| 1 \| \|  \| A0A248ZY02 \| Large ribosomal subunit protein uL3 OS=Mannheimia haemolytica OX=75985 GN=rplC PE=3 SV=1 \| 19 \| 2 \| 2 \| 2 \| 209 \| 22.5 \| 9.86 \| 4.4 \| 2 \| 1 \| \|  \| A0A3S4XDY6 \| Nucleoside permease OS=Mannheimia haemolytica OX=75985 GN=nupX_2 PE=3 SV=1 \| 5 \| 1 \| 1 \| 1 \| 420 \| 43.7 \| 7.18 \| 4.33 \| 1 \| 1 \| \|  \| A0A448T720 \| D-allose-binding periplasmic protein OS=Mannheimia haemolytica OX=75985 GN=alsB PE=4 SV=1 \| 5 \| 1 \| 1 \| 1 \| 315 \| 33 \| 5.94 \| 4.33 \| 1 \| 1 \| \|  \| A0A448T689 \| Uridine phosphorylase OS=Mannheimia haemolytica OX=75985 GN=udp PE=3 SV=1 \| 3 \| 1 \| 2 \| 1 \| 253 \| 27.4 \| 6.13 \| 4.3 \| 1 \| 1 \| \|  \| A0A448T6G4 \| Protein of uncharacterized function (DUF533) OS=Mannheimia haemolytica OX=75985 GN=NCTC10643_00535 PE=4 SV=1 \| 8 \| 1 \| 1 \| 1 \| 209 \| 21.9 \| 4.61 \| 4.28 \| 1 \| 1 \| \|  \| A0A448T7Z7 \| 5-deoxy-glucuronate isomerase OS=Mannheimia haemolytica OX=75985 GN=iolB PE=4 SV=1 \| 9 \| 1 \| 1 \| 1 \| 274 \| 31.5 \| 5.47 \| 4.2 \| 1 \| 1 \| \|  \| A0A3S4XLY7 \| Inosose dehydratase OS=Mannheimia haemolytica OX=75985 GN=iolE PE=3 SV=1 \| 6 \| 1 \| 1 \| 1 \| 298 \| 33.7 \| 5.39 \| 4.19 \| 1 \| 1 \| \|  \| A0A448T3L7 \| DNA starvation/stationary phase protection protein Dps OS=Mannheimia haemolytica OX=75985 GN=NCTC10643_00195 PE=3 SV=1 \| 10 \| 1 \| 1 \| 1 \| 168 \| 18.8 \| 4.94 \| 4.19 \| 1 \| 1 \| \|  \| A0A448T6W4 \| Heme/hemopexin utilization protein C OS=Mannheimia haemolytica OX=75985 GN=hxuC PE=3 SV=1 \| 2 \| 1 \| 1 \| 1 \| 701 \| 76.7 \| 9.25 \| 4.14 \| 1 \| 1 \| \|  \| A0A3S4XEL3 \| Lysine--tRNA ligase OS=Mannheimia haemolytica OX=75985 GN=lysS PE=3 SV=1 \| 4 \| 1 \| 1 \| 1 \| 500 \| 56.8 \| 5.02 \| 4.09 \| 1 \| 1 \| \|  \| A0A3S4XF89 \| Predicted metalloprotease OS=Mannheimia haemolytica OX=75985 GN=NCTC10643_01407 PE=4 SV=1 \| 10 \| 1 \| 1 \| 1 \| 279 \| 30.4 \| 7.42 \| 4.01 \| 1 \| 1 \| \|  \| A0A3S4XG90 \| Aromatic amino acid permease OS=Mannheimia haemolytica OX=75985 GN=tyrP PE=3 SV=1 \| 4 \| 1 \| 1 \| 1 \| 395 \| 42.1 \| 9.36 \| 4 \| 1 \| 1 \| \|  \| A0A3S5F3J4 \| Lipoprotein NlpI OS=Mannheimia haemolytica OX=75985 GN=nlpI PE=4 SV=1 \| 8 \| 2 \| 2 \| 2 \| 307 \| 35.8 \| 5.03 \| 3.99 \| 2 \| 1 \| \|  \| A0A448T3D9 \| Murein hydrolase activator NlpD OS=Mannheimia haemolytica OX=75985 GN=nlpD_2 PE=4 SV=1 \| 7 \| 2 \| 2 \| 2 \| 426 \| 44.7 \| 8.95 \| 3.98 \| 2 \| 1 \| \|  \| A0A448T8N1 \| Ribose/galactose/methyl galactoside import ATP-binding protein OS=Mannheimia haemolytica OX=75985 GN=mglA_2 PE=3 SV=1 \| 4 \| 1 \| 1 \| 1 \| 499 \| 55.5 \| 7.11 \| 3.98 \| 1 \| 1 \| \|  \| A0A3S5B9E7 \| ATP synthase subunit delta OS=Mannheimia haemolytica OX=75985 GN=atpH PE=3 SV=1 \| 9 \| 1 \| 1 \| 1 \| 177 \| 19.4 \| 4.79 \| 3.96 \| 1 \| 1 \| \|  \| A0A3S5B6X6 \| Spermidine/putrescine import ATP-binding protein PotA OS=Mannheimia haemolytica OX=75985 GN=potA_2 PE=3 SV=1 \| 4 \| 1 \| 1 \| 1 \| 371 \| 41.7 \| 4.94 \| 3.92 \| 1 \| 1 \| \|  \| A0A3S5B364 \| Aquaglyceroporin OS=Mannheimia haemolytica OX=75985 GN=glpF PE=3 SV=1 \| 7 \| 1 \| 1 \| 1 \| 261 \| 27.2 \| 6.76 \| 3.9 \| 1 \| 1 \| \|  \| A0A3S5F3G4 \| Glutathione S-transferase GstB OS=Mannheimia haemolytica OX=75985 GN=gstB PE=4 SV=1 \| 7 \| 1 \| 1 \| 1 \| 205 \| 23.3 \| 6.33 \| 3.87 \| 1 \| 1 \| \|  \| A0A3S4XGH2 \| Alpha-1,4 glucan phosphorylase OS=Mannheimia haemolytica OX=75985 GN=malP_2 PE=3 SV=1 \| 2 \| 1 \| 1 \| 1 \| 789 \| 90.5 \| 6.65 \| 3.87 \| 1 \| 1 \| \|  \| A0A3S4XBJ0 \| Ribosomal protein L11 methyltransferase OS=Mannheimia haemolytica OX=75985 GN=prmA PE=3 SV=1 \| 6 \| 1 \| 1 \| 1 \| 293 \| 32.2 \| 4.48 \| 3.86 \| 1 \| 1 \| \|  \| A0A448TBN7 \| Acetyl-coenzyme A carboxylase carboxyl transferase subunit beta OS=Mannheimia haemolytica OX=75985 GN=accD PE=3 SV=1 \| 5 \| 1 \| 1 \| 1 \| 298 \| 32.7 \| 7.36 \| 3.85 \| 1 \| 1 \| \|  \| A0A3S4WX39 \| 16S rRNA (cytosine(967)-C(5))-methyltransferase OS=Mannheimia haemolytica OX=75985 GN=rsmB PE=3 SV=1 \| 3 \| 1 \| 1 \| 1 \| 448 \| 50.5 \| 8.51 \| 3.79 \| 1 \| 1 \| \|  \| A0A248ZYP7 \| Small ribosomal subunit protein uS2 OS=Mannheimia haemolytica OX=75985 GN=rpsB PE=3 SV=1 \| 6 \| 1 \| 1 \| 1 \| 239 \| 26.4 \| 6.81 \| 3.74 \| 1 \| 1 \| \|  \| A0A448TCV7 \| Glycogen synthase OS=Mannheimia haemolytica OX=75985 GN=glgA PE=3 SV=1 \| 4 \| 1 \| 1 \| 1 \| 514 \| 57.8 \| 7.5 \| 3.73 \| 1 \| 1 \| \|  \| A0A3S4WYU8 \| Peptidyl-prolyl cis-trans isomerase D OS=Mannheimia haemolytica OX=75985 GN=ppiD PE=4 SV=1 \| 4 \| 1 \| 1 \| 1 \| 623 \| 69.5 \| 5.06 \| 3.71 \| 1 \| 1 \| \|  \| A0A3S4WZY7 \| Hypoxanthine phosphoribosyltransferase OS=Mannheimia haemolytica OX=75985 GN=hpt PE=3 SV=1 \| 9 \| 1 \| 1 \| 1 \| 179 \| 20.4 \| 5.33 \| 3.7 \| 1 \| 1 \| \|  \| A0A3S4X0H2 \| S-adenosylmethionine synthase OS=Mannheimia haemolytica OX=75985 GN=metK PE=3 SV=1 \| 5 \| 1 \| 1 \| 1 \| 383 \| 41.7 \| 5.72 \| 3.7 \| 1 \| 1 \| \|  \| A0A448T3L5 \| Beta-ketoacyl-[acyl-carrier-protein] synthase III OS=Mannheimia haemolytica OX=75985 GN=fabH PE=3 SV=1 \| 7 \| 1 \| 1 \| 1 \| 316 \| 34.1 \| 4.96 \| 3.65 \| 1 \| 1 \| \|  \| A0A448TB35 \| oxoglutarate dehydrogenase (succinyl-transferring) OS=Mannheimia haemolytica OX=75985 GN=sucA PE=3 SV=1 \| 3 \| 1 \| 1 \| 1 \| 936 \| 106.6 \| 6.29 \| 3.64 \| 1 \| 1 \| \|  \| A0A249A2J5 \| Na(+)/H(+) antiporter NhaA OS=Mannheimia haemolytica OX=75985 GN=nhaA PE=3 SV=1 \| 5 \| 1 \| 1 \| 1 \| 396 \| 42.4 \| 6.39 \| 3.61 \| 1 \| 1 \| \|  \| A0A3S5B7E9 \| Triosephosphate isomerase OS=Mannheimia haemolytica OX=75985 GN=tpiA PE=3 SV=1 \| 6 \| 1 \| 1 \| 1 \| 263 \| 27.5 \| 6.01 \| 3.49 \| 1 \| 1 \| \|  \| A0A3S4XDD9 \| Orotate phosphoribosyltransferase OS=Mannheimia haemolytica OX=75985 GN=pyrE PE=3 SV=1 \| 7 \| 1 \| 1 \| 1 \| 213 \| 23.6 \| 5.71 \| 3.49 \| 1 \| 1 \| \|  \| A0A3S5F333 \| Glutathione biosynthesis bifunctional protein GshAB OS=Mannheimia haemolytica OX=75985 GN=gshAB PE=3 SV=1 \| 2 \| 1 \| 1 \| 1 \| 758 \| 85.3 \| 5.45 \| 3.44 \| 1 \| 1 \| \|  \| A0A3S4XCT2 \| Probable L-asparaginase OS=Mannheimia haemolytica OX=75985 GN=ansA PE=3 SV=1 \| 8 \| 1 \| 1 \| 1 \| 321 \| 35 \| 6.87 \| 3.36 \| 1 \| 1 \| \|  \| A0A3S4WV38 \| PTS system mannose-specific EIID component OS=Mannheimia haemolytica OX=75985 GN=manZ PE=4 SV=1 \| 7 \| 1 \| 1 \| 1 \| 278 \| 30.2 \| 9.41 \| 3.29 \| 1 \| 1 \| \|  \| A0A3S4X0P6 \| Protein FdhE homolog OS=Mannheimia haemolytica OX=75985 GN=fdhE PE=3 SV=1 \| 5 \| 1 \| 1 \| 1 \| 305 \| 34.5 \| 5.24 \| 3.29 \| 1 \| 1 \| \|  \| A0A3S4Z5C1 \| Phosphatase YniC OS=Mannheimia haemolytica OX=75985 GN=yniC PE=4 SV=1 \| 8 \| 1 \| 1 \| 1 \| 215 \| 23.9 \| 5.2 \| 3.28 \| 1 \| 1 \| \|  \| A0A1D2Q7R7 \| Hybrid peroxiredoxin hyPrx5 OS=Mannheimia haemolytica OX=75985 GN=NCTC10643_00832 PE=4 SV=1 \| 7 \| 1 \| 1 \| 1 \| 244 \| 26.9 \| 5.35 \| 3.2 \| 1 \| 1 \| \|  \| A0A3S4XWV0 \| Oxaloacetate decarboxylase beta chain OS=Mannheimia haemolytica OX=75985 GN=oadB PE=3 SV=1 \| 6 \| 1 \| 1 \| 1 \| 434 \| 45.6 \| 7.53 \| 3.15 \| 1 \| 1 \| \|  \| A0A448TC04 \| Phospho-2-dehydro-3-deoxyheptonate aldolase OS=Mannheimia haemolytica OX=75985 GN=aroG PE=3 SV=1 \| 6 \| 1 \| 1 \| 1 \| 360 \| 39 \| 6.47 \| 3.15 \| 1 \| 1 \| \|  \| A0A1D2Q7T2 \| Glycerol-3-phosphate regulon repressor OS=Mannheimia haemolytica OX=75985 GN=glpR_1 PE=4 SV=1 \| 8 \| 1 \| 1 \| 1 \| 253 \| 28.3 \| 5.11 \| 3.15 \| 1 \| 1 \| \|  \| A0A448TBV2 \| Translation initiation factor IF-2 OS=Mannheimia haemolytica OX=75985 GN=infB PE=3 SV=1 \| 2 \| 1 \| 1 \| 1 \| 844 \| 91.9 \| 6.73 \| 3.13 \| 1 \| 1 \| \|  \| A0A448TCY5 \| Outer membrane protein 26 OS=Mannheimia haemolytica OX=75985 GN=NCTC10643_01662 PE=3 SV=1 \| 12 \| 1 \| 1 \| 1 \| 263 \| 29.2 \| 8.48 \| 3.12 \| 1 \| 1 \| \|  \| A0A3S4Z7Q6 \| ribonucleoside-diphosphate reductase OS=Mannheimia haemolytica OX=75985 GN=nrdB_1 PE=3 SV=1 \| 5 \| 1 \| 1 \| 1 \| 376 \| 43.7 \| 4.92 \| 3.07 \| 1 \| 1 \| \|  \| A0A448T6I1 \| G-3-P permease OS=Mannheimia haemolytica OX=75985 GN=glpT_2 PE=3 SV=1 \| 4 \| 1 \| 1 \| 1 \| 481 \| 53.4 \| 8.59 \| 3.04 \| 1 \| 1 \| \|  \| A0A3S4XL44 \| Aerobic glycerol-3-phosphate dehydrogenase OS=Mannheimia haemolytica OX=75985 GN=glpD PE=3 SV=1 \| 3 \| 1 \| 1 \| 1 \| 531 \| 58.8 \| 7.28 \| 3.01 \| 1 \| 1 \| \|  \| A0A3S4XP21 \| Ribonucleoside-diphosphate reductase OS=Mannheimia haemolytica OX=75985 GN=nrdA PE=3 SV=1 \| 3 \| 1 \| 1 \| 1 \| 756 \| 85.5 \| 6.51 \| 3.01 \| 1 \| 1 \| \|  \| A0A3S5B2D9 \| Mannonate dehydratase OS=Mannheimia haemolytica OX=75985 GN=uxuA PE=3 SV=1 \| 6 \| 1 \| 1 \| 1 \| 394 \| 44.7 \| 5.43 \| 3.01 \| 1 \| 1 \| \|  \| A0A448T6J7 \| Bacterial protein of uncharacterized function (DUF883) OS=Mannheimia haemolytica OX=75985 GN=NCTC10643_00545 PE=3 SV=1 \| 17 \| 1 \| 1 \| 1 \| 103 \| 11.6 \| 5.36 \| 3.01 \| 1 \| 1 \| \|  \| A0A448T8D1 \| 2-succinyl-5-enolpyruvyl-6-hydroxy-3-cyclohexene-1-carboxylate synthase OS=Mannheimia haemolytica OX=75985 GN=menD PE=3 SV=1 \| 5 \| 1 \| 1 \| 1 \| 568 \| 63.1 \| 6.93 \| 2.97 \| 1 \| 1 \| \|  \| A0A448TBQ4 \| Fructose-1,6-bisphosphatase OS=Mannheimia haemolytica OX=75985 GN=glpX PE=3 SV=1 \| 5 \| 1 \| 1 \| 1 \| 339 \| 36.7 \| 5.71 \| 2.94 \| 1 \| 1 \| \|  \| A0A3S4XGA0 \| Ribonucleoside-diphosphate reductase subunit alpha OS=Mannheimia haemolytica OX=75985 GN=nrdE PE=3 SV=1 \| 4 \| 1 \| 1 \| 1 \| 555 \| 63.1 \| 5.95 \| 2.91 \| 1 \| 1 \| \|  \| A0A3S4WXR5 \| 3D-(3,5/4)-trihydroxycyclohexane-1,2-dione hydrolase OS=Mannheimia haemolytica OX=75985 GN=iolD PE=3 SV=1 \| 2 \| 1 \| 1 \| 1 \| 644 \| 70.3 \| 6.32 \| 2.87 \| 1 \| 1 \| \|  \| A0A448TBM9 \| Hemolysin transporter protein shlB OS=Mannheimia haemolytica OX=75985 GN=shlB PE=4 SV=1 \| 3 \| 1 \| 1 \| 1 \| 589 \| 66 \| 8.44 \| 2.86 \| 1 \| 1 \| \|  \| A0A448TCN7 \| Pseudo-HPr OS=Mannheimia haemolytica OX=75985 GN=fruB PE=4 SV=1 \| 4 \| 1 \| 1 \| 1 \| 492 \| 51.6 \| 5.41 \| 2.83 \| 1 \| 1 \| \|  \| A0A3S4WZW7 \| Stringent starvation protein A homolog OS=Mannheimia haemolytica OX=75985 GN=sspA PE=3 SV=1 \| 7 \| 1 \| 1 \| 1 \| 214 \| 24.5 \| 5.31 \| 2.83 \| 1 \| 1 \| \|  \| A0A448T631 \| Peptidyl-prolyl cis-trans isomerase OS=Mannheimia haemolytica OX=75985 GN=fklB PE=3 SV=1 \| 9 \| 1 \| 1 \| 1 \| 209 \| 22.2 \| 4.5 \| 2.77 \| 1 \| 1 \| \|  \| A0A3S5B6J1 \| AMP nucleosidase OS=Mannheimia haemolytica OX=75985 GN=ygdH PE=4 SV=1 \| 3 \| 1 \| 1 \| 1 \| 455 \| 51 \| 6.6 \| 2.74 \| 1 \| 1 \| \|  \| A0A448T267 \| FAD:protein FMN transferase OS=Mannheimia haemolytica OX=75985 GN=apbE PE=3 SV=1 \| 6 \| 1 \| 1 \| 1 \| 344 \| 37.9 \| 6.58 \| 2.73 \| 1 \| 1 \| \|  \| A0A3S4XEG5 \| Elongation factor 4 OS=Mannheimia haemolytica OX=75985 GN=lepA PE=3 SV=1 \| 3 \| 1 \| 1 \| 1 \| 599 \| 66.3 \| 5.52 \| 2.69 \| 1 \| 1 \| \|  \| A0A3S4XNL0 \| pre-crRNA processing endonuclease OS=Mannheimia haemolytica OX=75985 GN=NCTC10643_01214 PE=3 SV=1 \| 6 \| 1 \| 1 \| 1 \| 225 \| 25.8 \| 7.84 \| 2.69 \| 1 \| 1 \| \|  \| A0A3S4Z8P1 \| Adenine phosphoribosyltransferase OS=Mannheimia haemolytica OX=75985 GN=apt PE=3 SV=1 \| 11 \| 1 \| 1 \| 1 \| 179 \| 19.5 \| 6.35 \| 2.69 \| 1 \| 1 \| \|  \| A0A448T807 \| Alpha-L-fucosidase OS=Mannheimia haemolytica OX=75985 GN=NCTC10643_00740 PE=4 SV=1 \| 5 \| 1 \| 1 \| 1 \| 446 \| 51.4 \| 5.68 \| 2.69 \| 1 \| 1 \| \|  \| A0A3S4X738 \| Na(+)-translocating NADH-quinone reductase subunit C OS=Mannheimia haemolytica OX=75985 GN=nqrC PE=3 SV=1 \| 4 \| 1 \| 1 \| 1 \| 257 \| 27.4 \| 6.6 \| 2.63 \| 1 \| 1 \| \|  \| A0A3S4XTI7 \| Phosphatidylserine decarboxylase proenzyme OS=Mannheimia haemolytica OX=75985 GN=psd PE=3 SV=1 \| 5 \| 1 \| 1 \| 1 \| 296 \| 33.2 \| 6.95 \| 2.61 \| 1 \| 1 \| \|  \| A0A1D2Q7J6 \| Phosphoenolpyruvate-protein phosphotransferase OS=Mannheimia haemolytica OX=75985 GN=ptsI PE=3 SV=1 \| 4 \| 1 \| 1 \| 1 \| 573 \| 63.1 \| 4.88 \| 2.59 \| 1 \| 1 \| \|  \| A0A248ZWK1 \| ADP-L-glycero-D-manno-heptose-6-epimerase OS=Mannheimia haemolytica OX=75985 GN=rfaD PE=3 SV=1 \| 4 \| 1 \| 1 \| 1 \| 308 \| 34.6 \| 5.22 \| 2.57 \| 1 \| 1 \| \|  \| A0A3S5B2G3 \| TPR repeat-containing protein NMB0313 OS=Mannheimia haemolytica OX=75985 GN=NCTC10643_00491 PE=3 SV=1 \| 2 \| 1 \| 1 \| 1 \| 483 \| 56.1 \| 9.41 \| 2.53 \| 1 \| 1 \| \|  \| A0A3S5B663 \| Glucosamine--fructose-6-phosphate aminotransferase OS=Mannheimia haemolytica OX=75985 GN=NCTC10643_01610 PE=4 SV=1 \| 7 \| 1 \| 1 \| 1 \| 266 \| 29.9 \| 6.89 \| 2.53 \| 1 \| 1 \| \|  \| A0A448TB41 \| ATP-dependent RNA helicase RhlB OS=Mannheimia haemolytica OX=75985 GN=rhlB PE=3 SV=1 \| 3 \| 1 \| 1 \| 1 \| 413 \| 46.7 \| 6.6 \| 2.53 \| 1 \| 1 \| \|  \| A0A448TEG4 \| Bifunctional aspartokinase/homoserine dehydrogenase OS=Mannheimia haemolytica OX=75985 GN=thrA PE=3 SV=1 \| 2 \| 1 \| 1 \| 1 \| 818 \| 88.2 \| 5.33 \| 2.5 \| 1 \| 1 \| \|  \| A0A448TCL6 \| Carboxy-S-adenosyl-L-methionine synthase OS=Mannheimia haemolytica OX=75985 GN=cmoA PE=3 SV=1 \| 7 \| 1 \| 1 \| 1 \| 242 \| 27.5 \| 5.85 \| 2.46 \| 1 \| 1 \| \|  \| A0A448T9L8 \| Arginine--tRNA ligase OS=Mannheimia haemolytica OX=75985 GN=argS PE=3 SV=1 \| 3 \| 1 \| 1 \| 1 \| 575 \| 64 \| 5.38 \| 2.43 \| 1 \| 1 \| \|  \| A0A3S4Z2X8 \| Protein involved in cell division OS=Mannheimia haemolytica OX=75985 GN=NCTC10643_00103 PE=4 SV=1 \| 10 \| 1 \| 1 \| 1 \| 246 \| 28.5 \| 5.87 \| 2.39 \| 1 \| 1 \| \|  \| A0A3S4XN53 \| Outer membrane protein W OS=Mannheimia haemolytica OX=75985 GN=ompW PE=4 SV=1 \| 5 \| 1 \| 1 \| 1 \| 223 \| 23.8 \| 9.47 \| 2.38 \| 1 \| 1 \| \|  \| A0A3S5F3G9 \| Bifunctional protein FolD OS=Mannheimia haemolytica OX=75985 GN=folD PE=3 SV=1 \| 4 \| 1 \| 1 \| 1 \| 286 \| 30.8 \| 6.73 \| 2.36 \| 1 \| 1 \| \|  \| A0A1D2Q656 \| Universal stress protein OS=Mannheimia haemolytica OX=75985 GN=uspA PE=3 SV=1 \| 12 \| 1 \| 1 \| 1 \| 141 \| 15.5 \| 4.7 \| 2.35 \| 1 \| 1 \| \|  \| A0A249A399 \| C4-dicarboxylate ABC transporter substrate-binding protein OS=Mannheimia haemolytica OX=75985 GN=CKG23_13840 PE=4 SV=1 \| 4 \| 1 \| 1 \| 1 \| 320 \| 34.5 \| 7.81 \| 2.35 \| 1 \| 1 \| \|  \| A0A3S4XZ78 \| Leukotoxin translocation ATP-binding protein LktB OS=Mannheimia haemolytica OX=75985 GN=gsiA_8 PE=4 SV=1 \| 3 \| 1 \| 1 \| 1 \| 531 \| 59.2 \| 6.32 \| 2.34 \| 1 \| 1 \| \|  \| A0A3S4Z5F0 \| Putative acyl-CoA dehydrogenase OS=Mannheimia haemolytica OX=75985 GN=NCTC10643_00633 PE=4 SV=1 \| 4 \| 1 \| 1 \| 1 \| 352 \| 38.8 \| 5.24 \| 2.34 \| 1 \| 1 \| \|  \| A0A448T6C3 \| Pyrimidine-specific ribonucleoside hydrolase rihB OS=Mannheimia haemolytica OX=75985 GN=rihB PE=4 SV=1 \| 7 \| 1 \| 1 \| 1 \| 310 \| 33.7 \| 5.6 \| 2.34 \| 1 \| 1 \| \|  \| A0A3S4XUJ2 \| Peptidyl-prolyl cis-trans isomerase OS=Mannheimia haemolytica OX=75985 GN=NCTC10643_00372 PE=3 SV=1 \| 4 \| 1 \| 1 \| 1 \| 241 \| 26.3 \| 8.07 \| 2.33 \| 1 \| 1 \| \|  \| A0A1D2Q774 \| NAD kinase OS=Mannheimia haemolytica OX=75985 GN=ppnK PE=3 SV=1 \| 4 \| 1 \| 1 \| 1 \| 294 \| 32.8 \| 6.23 \| 2.3 \| 1 \| 1 \| \|  \| A0A448T3T9 \| Branched-chain amino acid transport system carrier protein OS=Mannheimia haemolytica OX=75985 GN=brnQ PE=3 SV=1 \| 5 \| 1 \| 1 \| 1 \| 432 \| 46.4 \| 9.07 \| 2.24 \| 1 \| 1 \| \|  \| A0A249A0N7 \| Ribose-phosphate pyrophosphokinase OS=Mannheimia haemolytica OX=75985 GN=prs PE=3 SV=1 \| 4 \| 1 \| 1 \| 1 \| 316 \| 34.2 \| 6 \| 2.24 \| 1 \| 1 \| \|  \| A0A448TBC3 \| Efflux pump membrane transporter BepE OS=Mannheimia haemolytica OX=75985 GN=bepE PE=4 SV=1 \| 1 \| 1 \| 1 \| 1 \| 1055 \| 113.3 \| 5.45 \| 2.23 \| 1 \| 1 \| \|  \| A0A248ZWA7 \| Small ribosomal subunit protein uS3 OS=Mannheimia haemolytica OX=75985 GN=rpsC PE=3 SV=1 \| 6 \| 1 \| 1 \| 1 \| 235 \| 25.8 \| 10.17 \| 2.18 \| 1 \| 1 \| \|  \| A0A448T4M2 \| Succinyl-diaminopimelate desuccinylase OS=Mannheimia haemolytica OX=75985 GN=dapE PE=3 SV=1 \| 4 \| 1 \| 1 \| 1 \| 382 \| 41.4 \| 5.44 \| 2.17 \| 1 \| 1 \| \|  \| A0A249A2G8 \| Metal ABC transporter substrate-binding protein OS=Mannheimia haemolytica OX=75985 GN=CKG23_11485 PE=3 SV=1 \| 5 \| 1 \| 1 \| 1 \| 298 \| 33 \| 8.09 \| 2.13 \| 1 \| 1 \| \|  \| A0A3S5F384 \| Glycine betaine transporter BetP OS=Mannheimia haemolytica OX=75985 GN=betP PE=4 SV=1 \| 2 \| 1 \| 1 \| 1 \| 669 \| 75.3 \| 5.71 \| 2.13 \| 1 \| 1 \| \|  \| A0A249A0T7 \| Orotidine 5'-phosphate decarboxylase OS=Mannheimia haemolytica OX=75985 GN=pyrF PE=3 SV=1 \| 5 \| 1 \| 1 \| 1 \| 230 \| 25.4 \| 5.66 \| 2.12 \| 1 \| 1 \| \|  \| A0A448T8J1 \| Galactoside transport system permease protein mglC OS=Mannheimia haemolytica OX=75985 GN=mglC PE=4 SV=1 \| 4 \| 1 \| 1 \| 1 \| 336 \| 36.1 \| 8.85 \| 2.11 \| 1 \| 1 \| \|  \| A0A448TAZ7 \| Uncharacterized protein predicted to be involved in DNA repair OS=Mannheimia haemolytica OX=75985 GN=NCTC10643_01216 PE=4 SV=1 \| 4 \| 1 \| 1 \| 1 \| 287 \| 32.6 \| 6.02 \| 2.09 \| 1 \| 1 \| \|  \| A0A3S5B6E4 \| Deoxyguanosinetriphosphate triphosphohydrolase-like protein OS=Mannheimia haemolytica OX=75985 GN=dgt PE=3 SV=1 \| 4 \| 1 \| 1 \| 1 \| 441 \| 50.9 \| 6.83 \| 2 \| 1 \| 1 \| \|  \| A0A448T9G9 \| Predicted periplasmic/secreted protein OS=Mannheimia haemolytica OX=75985 GN=NCTC10643_00897 PE=4 SV=1 \| 6 \| 1 \| 1 \| 1 \| 239 \| 26.2 \| 6.42 \| 1.99 \| 1 \| 1 \| \|  \| A0A3S4XVM6 \| Transglutaminase-like superfamily OS=Mannheimia haemolytica OX=75985 GN=NCTC10643_00604 PE=4 SV=1 \| 4 \| 1 \| 1 \| 1 \| 368 \| 41 \| 5.4 \| 1.98 \| 1 \| 1 \| \|  \| A0A448T9L3 \| Probable protease sohB OS=Mannheimia haemolytica OX=75985 GN=sohB PE=3 SV=1 \| 4 \| 1 \| 1 \| 1 \| 350 \| 39.3 \| 7.3 \| 1.95 \| 1 \| 1 \| \|  \| A0A448T261 \| Argininosuccinate lyase OS=Mannheimia haemolytica OX=75985 GN=argH PE=3 SV=1 \| 3 \| 1 \| 1 \| 1 \| 459 \| 50.8 \| 5.35 \| 1.9 \| 1 \| 1 \| \| \| --- \| --- \| --- \| --- \| --- \| --- \| --- \| --- \| --- \| --- \| --- \| --- \| --- \| --- \| --- \| --- \| --- \| --- \| --- \| --- \| --- \| --- \| --- \| --- \| --- \| --- \| --- \| --- \| --- \| --- \| --- \| --- \| --- \| --- \| --- \| --- \| --- \| --- \| --- \| --- \| --- \| --- \| --- \| --- \| --- \| --- \| --- \| --- \| --- \| --- \| --- \| --- \| --- \| --- \| --- \| --- \| --- \| --- \| --- \| --- \| --- \| --- \| --- \| --- \| --- \| --- \| --- \| --- \| --- \| --- \| --- \| --- \| --- \| --- \| --- \| --- \| --- \| --- \| --- \| --- \| --- \| --- \| --- \| --- \| --- \| --- \| --- \| --- \| --- \| --- \| --- \| --- \| --- \| --- \| --- \| --- \| --- \| --- \| --- \| --- \| --- \| --- \| --- \| --- \| --- \| --- \| --- \| --- \| --- \| --- \| --- \| --- \| --- \| --- \| --- \| --- \| --- \| --- \| --- \| --- \| --- \| --- \| --- \| --- \| --- \| --- \| --- \| --- \| --- \| --- \| --- \| --- \| --- \| --- \| --- \| --- \| --- \| --- \| --- \| --- \| --- \| --- \| --- \| --- \| --- \| --- \| --- \| --- \| --- \| --- \| --- \| --- \| --- \| --- \| --- \| --- \| --- \| --- \| --- \| --- \| --- \| --- \| --- \| --- \| --- \| --- \| --- \| --- \| --- \| --- \| --- \| --- \| --- \| --- \| --- \| --- \| --- \| --- \| --- \| --- \| --- \| --- \| --- \| --- \| --- \| --- \| --- \| --- \| --- \| --- \| --- \| --- \| --- \| --- \| --- \| --- \| --- \| --- \| --- \| --- \| --- \| --- \| --- \| --- \| --- \| --- \| --- \| --- \| --- \| --- \| --- \| --- \| --- \| --- \| --- \| --- \| --- \| --- \| --- \| --- \| --- \| --- \| --- \| --- \| --- \| --- \| --- \| --- \| --- \| --- \| --- \| --- \| --- \| --- \| --- \| --- \| --- \| --- \| --- \| --- \| --- \| --- \| --- \| --- \| --- \| --- \| --- \| --- \| --- \| --- \| --- \| --- \| --- \| --- \| --- \| --- \| --- \| --- \| --- \| --- \| --- \| --- \| --- \| --- \| --- \| --- \| --- \| --- \| --- \| --- \| --- \| --- \| --- \| --- \| --- \| --- \| --- \| --- \| --- \| --- \| --- \| --- \| --- \| --- \| --- \| --- \| --- \| --- \| --- \| --- \| --- \| --- \| --- \| --- \| --- \| --- \| --- \| --- \| --- \| --- \| --- \| --- \| --- \| --- \| --- \| --- \| --- \| --- \| --- \| --- \| --- \| --- \| --- \| --- \| --- \| --- \| --- \| --- \| --- \| --- \| --- \| --- \| --- \| --- \| --- \| --- \| --- \| --- \| --- \| --- \| --- \| --- \| --- \| --- \| --- \| --- \| --- \| --- \| --- \| --- \| --- \| --- \| --- \| --- \| --- \| --- \| --- \| --- \| --- \| --- \| --- \| --- \| --- \| --- \| --- \| --- \| --- \| --- \| --- \| --- \| --- \| --- \| --- \| --- \| --- \| --- \| --- \| --- \| --- \| --- \| --- \| --- \| --- \| --- \| --- \| --- \| --- \| --- \| --- \| --- \| --- \| --- \| --- \| --- \| --- \| --- \| --- \| --- \| --- \| --- \| --- \| --- \| --- \| --- \| --- \| --- \| --- \| --- \| --- \| --- \| --- \| --- \| --- \| --- \| --- \| --- \| --- \| --- \| --- \| --- \| --- \| --- \| --- \| --- \| --- \| --- \| --- \| --- \| --- \| --- \| --- \| --- \| --- \| --- \| --- \| --- \| --- \| --- \| --- \| --- \| --- \| --- \| --- \| --- \| --- \| --- \| --- \| --- \| --- \| --- \| --- \| --- \| --- \| --- \| --- \| --- \| --- \| --- \| --- \| --- \| --- \| --- \| --- \| --- \| --- \| --- \| --- \| --- \| --- \| --- \| --- \| --- \| --- \| --- \| --- \| --- \| --- \| --- \| --- \| --- \| --- \| --- \| --- \| --- \| --- \| --- \| --- \| --- \| --- \| --- \| --- \| --- \| --- \| --- \| --- \| --- \| --- \| --- \| --- \| --- \| --- \| --- \| --- \| --- \| --- \| --- \| --- \| --- \| --- \| --- \| --- \| --- \| --- \| --- \| --- \| --- \| --- \| --- \| --- \| --- \| --- \| --- \| --- \| --- \| --- \| --- \| --- \| --- \| --- \| --- \| --- \| --- \| --- \| --- \| --- \| --- \| --- \| --- \| --- \| --- \| --- \| --- \| --- \| --- \| --- \| --- \| --- \| --- \| --- \| --- \| --- \| --- \| --- \| --- \| --- \| --- \| --- \| --- \| --- \| --- \| --- \| --- \| --- \| --- \| --- \| --- \| --- \| --- \| --- \| --- \| --- \| --- \| --- \| --- \| --- \| --- \| --- \| --- \| --- \| --- \| --- \| --- \| --- \| --- \| --- \| --- \| --- \| --- \| --- \| --- \| --- \| --- \| --- \| --- \| --- \| --- \| --- \| --- \| --- \| --- \| --- \| --- \| --- \| --- \| --- \| --- \| --- \| --- \| --- \| --- \| --- \| --- \| --- \| --- \| --- \| --- \| --- \| --- \| --- \| --- \| --- \| --- \| --- \| --- \| --- \| --- \| --- \| --- \| --- \| --- \| --- \| --- \| --- \| --- \| --- \| --- \| --- \| --- \| --- \| --- \| --- \| --- \| --- \| --- \| --- \| --- \| --- \| --- \| --- \| --- \| --- \| --- \| --- \| --- \| --- \| --- \| --- \| --- \| --- \| --- \| --- \| --- \| --- \| --- \| --- \| --- \| --- \| --- \| --- \| --- \| --- \| --- \| --- \| --- \| --- \| --- \| --- \| --- \| --- \| --- \| --- \| --- \| --- \| --- \| --- \| --- \| --- \| --- \| --- \| --- \| --- \| --- \| --- \| --- \| --- \| --- \| --- \| --- \| --- \| --- \| --- \| --- \| --- \| --- \| --- \| --- \| --- \| --- \| --- \| --- \| --- \| --- \| --- \| --- \| --- \| --- \| --- \| --- \| --- \| --- \| --- \| --- \| --- \| --- \| --- \| --- \| --- \| --- \| --- \| --- \| --- \| --- \| --- \| --- \| --- \| --- \| --- \| --- \| --- \| --- \| --- \| --- \| --- \| --- \| --- \| --- \| --- \| --- \| --- \| --- \| --- \| --- \| --- \| --- \| --- \| --- \| --- \| --- \| --- \| --- \| --- \| --- \| --- \| --- \| --- \| --- \| --- \| --- \| --- \| --- \| --- \| --- \| --- \| --- \| --- \| --- \| --- \| --- \| --- \| --- \| --- \| --- \| --- \| --- \| --- \| --- \| --- \| --- \| --- \| --- \| --- \| --- \| --- \| --- \| --- \| --- \| --- \| --- \| --- \| --- \| --- \| --- \| --- \| --- \| --- \| --- \| --- \| --- \| --- \| --- \| --- \| --- \| --- \| --- \| --- \| --- \| --- \| --- \| --- \| --- \| --- \| --- \| --- \| --- \| --- \| --- \| --- \| --- \| --- \| --- \| --- \| --- \| --- \| --- \| --- \| --- \| --- \| --- \| --- \| --- \| --- \| --- \| --- \| --- \| --- \| --- \| --- \| --- \| --- \| --- \| --- \| --- \| --- \| --- \| --- \| --- \| --- \| --- \| --- \| --- \| --- \| --- \| --- \| --- \| --- \| --- \| --- \| --- \| --- \| --- \| --- \| --- \| --- \| --- \| --- \| --- \| --- \| --- \| --- \| --- \| --- \| --- \| --- \| --- \| --- \| --- \| --- \| --- \| --- \| --- \| --- \| --- \| --- \| --- \| --- \| --- \| --- \| --- \| --- \| --- \| --- \| --- \| --- \| --- \| --- \| --- \| --- \| --- \| --- \| --- \| --- \| --- \| --- \| --- \| --- \| --- \| --- \| --- \| --- \| --- \| --- \| --- \| --- \| --- \| --- \| --- \| --- \| --- \| --- \| --- \| --- \| --- \| --- \| --- \| --- \| --- \| --- \| --- \| --- \| --- \| --- \| --- \| --- \| --- \| --- \| --- \| --- \| --- \| --- \| --- \| --- \| --- \| --- \| --- \| --- \| --- \| --- \| --- \| --- \| --- \| --- \| --- \| --- \| --- \| --- \| --- \| --- \| --- \| --- \| --- \| --- \| --- \| --- \| --- \| --- \| --- \| --- \| --- \| --- \| --- \| --- \| --- \| --- \| --- \| --- \| --- \| --- \| --- \| --- \| --- \| --- \| --- \| --- \| --- \| --- \| --- \| --- \| --- \| --- \| --- \| --- \| --- \| --- \| --- \| --- \| --- \| --- \| --- \| --- \| --- \| --- \| --- \| --- \| --- \| --- \| --- \| --- \| --- \| --- \| --- \| --- \| --- \| --- \| --- \| --- \| --- \| --- \| --- \| --- \| --- \| --- \| --- \| --- \| --- \| --- \| --- \| --- \| --- \| --- \| --- \| --- \| --- \| --- \| --- \| --- \| --- \| --- \| --- \| --- \| --- \| --- \| --- \| --- \| --- \| --- \| --- \| --- \| --- \| --- \| --- \| --- \| --- \| --- \| --- \| --- \| --- \| --- \| --- \| --- \| --- \| --- \| --- \| --- \| --- \| --- \| --- \| --- \| --- \| --- \| --- \| --- \| --- \| --- \| --- \| --- \| --- \| --- \| --- \| --- \| --- \| --- \| --- \| --- \| --- \| --- \| --- \| --- \| --- \| --- \| --- \| --- \| --- \| --- \| --- \| --- \| --- \| --- \| --- \| --- \| --- \| --- \| --- \| --- \| --- \| --- \| --- \| --- \| --- \| --- \| --- \| --- \| --- \| --- \| --- \| --- \| --- \| --- \| --- \| --- \| --- \| --- \| --- \| --- \| --- \| --- \| --- \| --- \| --- \| --- \| --- \| --- \| --- \| --- \| --- \| --- \| --- \| --- \| --- \| --- \| --- \| --- \| --- \| --- \| --- \| --- \| --- \| --- \| --- \| --- \| --- \| --- \| --- \| --- \| --- \| --- \| --- \| --- \| --- \| --- \| --- \| --- \| --- \| --- \| --- \| --- \| --- \| --- \| --- \| --- \| --- \| --- \| --- \| --- \| --- \| --- \| --- \| --- \| --- \| --- \| --- \| --- \| --- \| --- \| --- \| --- \| --- \| --- \| --- \| --- \| --- \| --- \| --- \| --- \| --- \| --- \| --- \| --- \| --- \| --- \| --- \| --- \| --- \| --- \| --- \| --- \| --- \| --- \| --- \| --- \| --- \| --- \| --- \| --- \| --- \| --- \| --- \| --- \| --- \| --- \| --- \| --- \| --- \| --- \| --- \| --- \| --- \| --- \| --- \| --- \| --- \| --- \| --- \| --- \| --- \| --- \| --- \| --- \| --- \| --- \| --- \| --- \| --- \| --- \| --- \| --- \| --- \| --- \| --- \| --- \| --- \| --- \| --- \| --- \| --- \| --- \| --- \| --- \| --- \| --- \| --- \| --- \| --- \| --- \| --- \| --- \| --- \| --- \| --- \| --- \| --- \| --- \| --- \| --- \| --- \| --- \| --- \| --- \| --- \| --- \| --- \| --- \| --- \| --- \| --- \| --- \| --- \| --- \| --- \| --- \| --- \| --- \| --- \| --- \| --- \| --- \| --- \| --- \| --- \| --- \| --- \| --- \| --- \| --- \| --- \| --- \| --- \| --- \| --- \| --- \| --- \| --- \| --- \| --- \| --- \| --- \| --- \| --- \| --- \| --- \| --- \| --- \| --- \| --- \| --- \| --- \| --- \| --- \| --- \| --- \| --- \| --- \| --- \| --- \| --- \| --- \| --- \| --- \| --- \| --- \| --- \| --- \| --- \| --- \| --- \| --- \| --- \| --- \| --- \| --- \| --- \| --- \| --- \| --- \| --- \| --- \| --- \| --- \| --- \| --- \| --- \| --- \| --- \| --- \| --- \| --- \| --- \| --- \| --- \| --- \| --- \| --- \| --- \| --- \| --- \| --- \| --- \| --- \| --- \| --- \| --- \| --- \| --- \| --- \| --- \| --- \| --- \| --- \| --- \| --- \| --- \| --- \| --- \| --- \| --- \| --- \| --- \| --- \| --- \| --- \| --- \| --- \| --- \| --- \| --- \| --- \| --- \| --- \| --- \| --- \| --- \| --- \| --- \| --- \| --- \| --- \| --- \| --- \| --- \| --- \| --- \| --- \| --- \| --- \| --- \| --- \| --- \| --- \| --- \| --- \| --- \| --- \| --- \| --- \| --- \| --- \| --- \| --- \| --- \| --- \| --- \| --- \| --- \| --- \| --- \| --- \| --- \| --- \| --- \| --- \| --- \| --- \| --- \| --- \| --- \| --- \| --- \| --- \| --- \| --- \| --- \| --- \| --- \| --- \| --- \| --- \| --- \| --- \| --- \| --- \| --- \| --- \| --- \| --- \| --- \| --- \| --- \| --- \| --- \| --- \| --- \| --- \| --- \| --- \| --- \| --- \| --- \| --- \| --- \| --- \| --- \| --- \| --- \| --- \| --- \| --- \| --- \| --- \| --- \| --- \| --- \| --- \| --- \| --- \| --- \| --- \| --- \| --- \| --- \| --- \| --- \| --- \| --- \| --- \| --- \| --- \| --- \| --- \| --- \| --- \| --- \| --- \| --- \| --- \| --- \| --- \| --- \| --- \| --- \| --- \| --- \| --- \| --- \| --- \| --- \| --- \| --- \| --- \| --- \| --- \| --- \| --- \| --- \| --- \| --- \| --- \| --- \| --- \| --- \| --- \| --- \| --- \| --- \| --- \| --- \| --- \| --- \| --- \| --- \| --- \| --- \| --- \| --- \| --- \| --- \| --- \| --- \| --- \| --- \| --- \| --- \| --- \| --- \| --- \| --- \| --- \| --- \| --- \| --- \| --- \| --- \| --- \| --- \| --- \| --- \| --- \| --- \| --- \| --- \| --- \| --- \| --- \| --- \| --- \| --- \| --- \| --- \| --- \| --- \| --- \| --- \| --- \| --- \| --- \| --- \| --- \| --- \| --- \| --- \| --- \| --- \| --- \| --- \| --- \| --- \| --- \| --- \| --- \| --- \| --- \| --- \| --- \| --- \| --- \| --- \| --- \| --- \| --- \| --- \| --- \| --- \| --- \| --- \| --- \| --- \| --- \| --- \| --- \| --- \| --- \| --- \| --- \| --- \| --- \| --- \| --- \| --- \| --- \| --- \| --- \| --- \| --- \| --- \| --- \| --- \| --- \| --- \| --- \| --- \| --- \| --- \| --- \| --- \| --- \| --- \| --- \| --- \| --- \| --- \| --- \| --- \| --- \| --- \| --- \| --- \| --- \| --- \| --- \| --- \| --- \| --- \| --- \| --- \| --- \| --- \| --- \| --- \| --- \| --- \| --- \| --- \| --- \| --- \| --- \| --- \| --- \| --- \| --- \| --- \| --- \| --- \| --- \| --- \| --- \| --- \| --- \| --- \| --- \| --- \| --- \| --- \| --- \| --- \| --- \| --- \| --- \| --- \| --- \| --- \| --- \| --- \| --- \| --- \| --- \| --- \| --- \| --- \| --- \| --- \| --- \| --- \| --- \| --- \| --- \| --- \| --- \| --- \| --- \| --- \| --- \| --- \| --- \| --- \| --- \| --- \| --- \| --- \| --- \| --- \| --- \| --- \| --- \| --- \| --- \| --- \| --- \| --- \| --- \| --- \| --- \| --- \| --- \| --- \| --- \| --- \| --- \| --- \| --- \| --- \| --- \| --- \| --- \| --- \| --- \| --- \| --- \| --- \| --- \| --- \| --- \| --- \| --- \| --- \| --- \| --- \| --- \| --- \| --- \| --- \| --- \| --- \| --- \| --- \| --- \| --- \| --- \| --- \| --- \| --- \| --- \| --- \| --- \| --- \| --- \| --- \| --- \| --- \| --- \| --- \| --- \| --- \| --- \| --- \| --- \| --- \| --- \| --- \| --- \| --- \| --- \| --- \| --- \| --- \| --- \| --- \| --- \| --- \| --- \| --- \| --- \| --- \| --- \| --- \| --- \| --- \| --- \| --- \| --- \| --- \| --- \| --- \| --- \| --- \| --- \| --- \| --- \| --- \| --- \| --- \| --- \| --- \| --- \| --- \| --- \| --- \| --- \| --- \| --- \| --- \| --- \| --- \| --- \| --- \| --- \| --- \| --- \| --- \| --- \| --- \| --- \| --- \| --- \| --- \| --- \| --- \| --- \| --- \| --- \| --- \| --- \| --- \| --- \| --- \| --- \| --- \| --- \| --- \| --- \| --- \| --- \| --- \| --- \| --- \| --- \| --- \| --- \| --- \| --- \| --- \| --- \| --- \| --- \| --- \| --- \| --- \| --- \| --- \| --- \| --- \| --- \| --- \| --- \| --- \| --- \| --- \| --- \| --- \| --- \| --- \| --- \| --- \| --- \| --- \| --- \| --- \| --- \| --- \| --- \| --- \| --- \| --- \| --- \| --- \| --- \| --- \| --- \| --- \| --- \| --- \| --- \| --- \| --- \| --- \| --- \| --- \| --- \| --- \| --- \| --- \| --- \| --- \| --- \| --- \| --- \| --- \| --- \| --- \| --- \| --- \| --- \| --- \| --- \| --- \| --- \| --- \| --- \| --- \| --- \| --- \| --- \| --- \| --- \| --- \| --- \| --- \| --- \| --- \| --- \| --- \| --- \| --- \| --- \| --- \| --- \| --- \| --- \| --- \| --- \| --- \| --- \| --- \| --- \| --- \| --- \| --- \| --- \| --- \| --- \| --- \| --- \| --- \| --- \| --- \| --- \| --- \| --- \| --- \| --- \| --- \| --- \| --- \| --- \| --- \| --- \| --- \| --- \| --- \| --- \| --- \| --- \| --- \| --- \| --- \| --- \| --- \| --- \| --- \| --- \| --- \| --- \| --- \| --- \| --- \| --- \| --- \| --- \| --- \| --- \| --- \| --- \| --- \| --- \| --- \| --- \| --- \| --- \| --- \| --- \| --- \| --- \| --- \| --- \| --- \| --- \| --- \| --- \| --- \| --- \| --- \| --- \| --- \| --- \| --- \| --- \| --- \| --- \| --- \| --- \| --- \| --- \| --- \| --- \| --- \| --- \| --- \| --- \| --- \| --- \| --- \| --- \| --- \| --- \| --- \| --- \| --- \| --- \| --- \| --- \| --- \| --- \| --- \| --- \| --- \| --- \| --- \| --- \| --- \| --- \| --- \| --- \| --- \| --- \| --- \| --- \| --- \| --- \| --- \| --- \| --- \| --- \| --- \| --- \| --- \| --- \| --- \| --- \| --- \| --- \| --- \| --- \| --- \| --- \| --- \| --- \| --- \| --- \| --- \| --- \| --- \| --- \| --- \| --- \| --- \| --- \| --- \| --- \| --- \| --- \| --- \| --- \| --- \| --- \| --- \| --- \| --- \| --- \| --- \| --- \| --- \| --- \| --- \| --- \| --- \| --- \| --- \| --- \| --- \| --- \| --- \| --- \| --- \| --- \| --- \| --- \| --- \| --- \| --- \| --- \| --- \| --- \| --- \| --- \| --- \| --- \| --- \| --- \| --- \| --- \| --- \| --- \| --- \| --- \| --- \| --- \| --- \| --- \| --- \| --- \| --- \| --- \| --- \| --- \| --- \| --- \| --- \| --- \| --- \| --- \| --- \| --- \| --- \| --- \| --- \| --- \| --- \| --- \| --- \| --- \| --- \| --- \| --- \| --- \| --- \| --- \| --- \| --- \| --- \| --- \| --- \| --- \| --- \| --- \| --- \| --- \| --- \| --- \| --- \| --- \| --- \| --- \| --- \| --- \| --- \| --- \| --- \| --- \| --- \| --- \| --- \| --- \| --- \| --- \| --- \| --- \| --- \| --- \| --- \| --- \| --- \| --- \| --- \| --- \| --- \| --- \| --- \| --- \| --- \| --- \| --- \| --- \| --- \| --- \| --- \| --- \| --- \| --- \| --- \| --- \| --- \| --- \| --- \| --- \| --- \| --- \| --- \| --- \| --- \| --- \| --- \| --- \| --- \| --- \| --- \| --- \| --- \| --- \| --- \| --- \| --- \| --- \| --- \| --- \| --- \| --- \| --- \| --- \| --- \| --- \| --- \| --- \| --- \| --- \| --- \| --- \| --- \| --- \| --- \| --- \| --- \| --- \| --- \| --- \| --- \| --- \| --- \| --- \| --- \| --- \| --- \| --- \| --- \| --- \| --- \| --- \| --- \| --- \| --- \| --- \| --- \| --- \| --- \| --- \| --- \| --- \| --- \| --- \| --- \| --- \| --- \| --- \| --- \| --- \| --- \| --- \| --- \| --- \| --- \| --- \| --- \| --- \| --- \| --- \| --- \| --- \| --- \| --- \| --- \| --- \| --- \| --- \| --- \| --- \| --- \| --- \| --- \| --- \| --- \| --- \| --- \| --- \| --- \| --- \| --- \| --- \| --- \| --- \| --- \| --- \| --- \| --- \| --- \| --- \| --- \| --- \| --- \| --- \| --- \| --- \| --- \| --- \| --- \| --- \| --- \| --- \| --- \| --- \| --- \| --- \| --- \| --- \| --- \| --- \| --- \| --- \| --- \| --- \| --- \| --- \| --- \| --- \| --- \| --- \| --- \| --- \| --- \| --- \| --- \| --- \| --- \| --- \| --- \| --- \| --- \| --- \| --- \| --- \| --- \| --- \| --- \| --- \| --- \| --- \| --- \| --- \| --- \| --- \| --- \| --- \| --- \| --- \| --- \| --- \| --- \| --- \| --- \| --- \| --- \| --- \| --- \| --- \| --- \| --- \| --- \| --- \| --- \| --- \| --- \| --- \| --- \| --- \| --- \| --- \| --- \| --- \| --- \| --- \| --- \| --- \| --- \| --- \| --- \| --- \| --- \| --- \| --- \| --- \| --- \| --- \| --- \| --- \| --- \| --- \| --- \| --- \| --- \| --- \| --- \| --- \| --- \| --- \| --- \| --- \| --- \| --- \| --- \| --- \| --- \| --- \| --- \| --- \| --- \| --- \| --- \| --- \| --- \| --- \| --- \| --- \| --- \| --- \| --- \| --- \| --- \| --- \| --- \| --- \| --- \| --- \| --- \| --- \| --- \| --- \| --- \| --- \| --- \| --- \| --- \| --- \| --- \| --- \| --- \| --- \| --- \| --- \| --- \| --- \| --- \| --- \| --- \| --- \| --- \| --- \| --- \| --- \| --- \| --- \| --- \| --- \| --- \| --- \| --- \| --- \| --- \| --- \| --- \| --- \| --- \| --- \| --- \| --- \| --- \| --- \| --- \| --- \| --- \| --- \| --- \| --- \| --- \| --- \| --- \| --- \| --- \| --- \| --- \| --- \| --- \| --- \| --- \| --- \| --- \| --- \| --- \| --- \| --- \| --- \| --- \| --- \| --- \| --- \| --- \| --- \| --- \| --- \| --- \| --- \| --- \| --- \| --- \| --- \| --- \| --- \| --- \| --- \| --- \| --- \| --- \| --- \| --- \| --- \| --- \| --- \| --- \| --- \| --- \| --- \| --- \| --- \| --- \| --- \| --- \| --- \| --- \| --- \| --- \| --- \| --- \| --- \| --- \| --- \| --- \| --- \| --- \| --- \| --- \| --- \| --- \| --- \| --- \| --- \| --- \| --- \| --- \| --- \| --- \| --- \| --- \| --- \| --- \| --- \| --- \| --- \| --- \| --- \| --- \| --- \| --- \| --- \| --- \| --- \| --- \| --- \| --- \| --- \| --- \| --- \| --- \| --- \| --- \| --- \| --- \| --- \| --- \| --- \| --- \| --- \| --- \| --- \| --- \| --- \| --- \| --- \| --- \| --- \| --- \| --- \| --- \| --- \| --- \| --- \| --- \| --- \| --- \| --- \| --- \| --- \| --- \| --- \| --- \| --- \| --- \| --- \| --- \| --- \| --- \| --- \| --- \| --- \| --- \| --- \| --- \| --- \| --- \| --- \| --- \| --- \| --- \| --- \| --- \| --- \| --- \| --- \| --- \| --- \| --- \| --- \| --- \| --- \| --- \| --- \| --- \| --- \| --- \| --- \| --- \| --- \| --- \| --- \| --- \| --- \| --- \| --- \| --- \| --- \| --- \| --- \| --- \| --- \| --- \| --- \| --- \| --- \| --- \| --- \| --- \| --- \| --- \| --- \| --- \| --- \| --- \| --- \| --- \| --- \| --- \| --- \| --- \| --- \| --- \| --- \| --- \| --- \| --- \| --- \| --- \| --- \| --- \| --- \| --- \| --- \| --- \| --- \| --- \| --- \| --- \| --- \| --- \| --- \| --- \| --- \| --- \| --- \| --- \| --- \| --- \| --- \| --- \| --- \| --- \| --- \| --- \| --- \| --- \| --- \| --- \| --- \| --- \| --- \| --- \| --- \| --- \| --- \| --- \| --- \| --- \| --- \| --- \| --- \| --- \| --- \| --- \| --- \| --- \| --- \| --- \| --- \| --- \| --- \| --- \| --- \| --- \| --- \| --- \| --- \| --- \| --- \| --- \| --- \| --- \| --- \| --- \| --- \| --- \| --- \| --- \| --- \| --- \| --- \| --- \| --- \| --- \| --- \| --- \| --- \| --- \| --- \| --- \| --- \| --- \| --- \| --- \| --- \| --- \| --- \| --- \| --- \| --- \| --- \| --- \| --- \| --- \| --- \| --- \| --- \| --- \| --- \| --- \| --- \| --- \| --- \| --- \| --- \| --- \| --- \| --- \| --- \| --- \| --- \| --- \| --- \| --- \| --- \| --- \| --- \| --- \| --- \| --- \| --- \| --- \| --- \| --- \| --- \| --- \| --- \| --- \| --- \| --- \| --- \| --- \| --- \| --- \| --- \| --- \| --- \| --- \| --- \| --- \| --- \| --- \| --- \| --- \| --- \| --- \| --- \| --- \| --- \| --- \| --- \| --- \| --- \| --- \| --- \| --- \| --- \| --- \| --- \| --- \| --- \| --- \| --- \| --- \| --- \| --- \| --- \| --- \| --- \| --- \| --- \| --- \| --- \| --- \| --- \| --- \| --- \| --- \| --- \| --- \| --- \| --- \| --- \| --- \| --- \| --- \| --- \| --- \| --- \| --- \| --- \| --- \| --- \| --- \| --- \| --- \| --- \| --- \| --- \| --- \| --- \| --- \| --- \| --- \| --- \| --- \| --- \| --- \| --- \| --- \| --- \| --- \| --- \| --- \| --- \| --- \| --- \| --- \| --- \| --- \| --- \| --- \| --- \| --- \| --- \| --- \| --- \| --- \| --- \| --- \| --- \| --- \| --- \| --- \| --- \| --- \| --- \| --- \| --- \| --- \| --- \| --- \| --- \| --- \| --- \| --- \| --- \| --- \| --- \| --- \| --- \| --- \| --- \| --- \| --- \| --- \| --- \| --- \| --- \| --- \| --- \| --- \| --- \| --- \| --- \| --- \| --- \| --- \| --- \| --- \| --- \| --- \| --- \| --- \| --- \| --- \| --- \| --- \| --- \| --- \| --- \| --- \| --- \| --- \| --- \| --- \| --- \| --- \| --- \| --- \| --- \| --- \| --- \| --- \| --- \| --- \| --- \| --- \| --- \| --- \| --- \| --- \| --- \| --- \| --- \| --- \| --- \| --- \| --- \| --- \| --- \| --- \| --- \| --- \| --- \| --- \| --- \| --- \| --- \| --- \| --- \| --- \| --- \| --- \| --- \| --- \| --- \| --- \| --- \| --- \| --- \| --- \| --- \| --- \| --- \| --- \| --- \| --- \| --- \| --- \| --- \| --- \| --- \| --- \| --- \| --- \| --- \| --- \| --- \| --- \| --- \| --- \| --- \| --- \| --- \| --- \| --- \| --- \| --- \| --- \| --- \| --- \| --- \| --- \| --- \| --- \| --- \| --- \| --- \| --- \| --- \| --- \| --- \| --- \| --- \| --- \| --- \| --- \| --- \| --- \| --- \| --- \| --- \| --- \| --- \| --- \| --- \| --- \| --- \| --- \| --- \| --- \| --- \| --- \| --- \| --- \| --- \| --- \| --- \| --- \| --- \| --- \| --- \| --- \| --- \| --- \| --- \| --- \| --- \| --- \| --- \| --- \| --- \| --- \| --- \| --- \| --- \| --- \| --- \| --- \| --- \| --- \| --- \| --- \| --- \| --- \| --- \| --- \| --- \| --- \| --- \| --- \| --- \| --- \| --- \| --- \| --- \| --- \| --- \| --- \| --- \| --- \| --- \| --- \| --- \| --- \| --- \| --- \| --- \| --- \| --- \| --- \| --- \| --- \| --- \| --- \| --- \| --- \| --- \| --- \| --- \| --- \| --- \| --- \| --- \| --- \| --- \| --- \| --- \| --- \| --- \| --- \| --- \| --- \| --- \| --- \| --- \| --- \| --- \| --- \| --- \| --- \| --- \| --- \| --- \| --- \| --- \| --- \| --- \| --- \| --- \| --- \| --- \| --- \| --- \| --- \| --- \| --- \| --- \| --- \| --- \| --- \| --- \| --- \| --- \| --- \| --- \| --- \| --- \| --- \| --- \| --- \| --- \| --- \| --- \| --- \| --- \| --- \| --- \| --- \| --- \| --- \| --- \| --- \| --- \| --- \| --- \| --- \| --- \| --- \| --- \| --- \| --- \| --- \| --- \| --- \| --- \| --- \| --- \| --- \| --- \| --- \| --- \| --- \| --- \| --- \| --- \| --- \| --- \| --- \| --- \| --- \| --- \| --- \| --- \| --- \| --- \| --- \| --- \| --- \| --- \| --- \| --- \| --- \| --- \| --- \| --- \| --- \| --- \| --- \| --- \| --- \| --- \| --- \| --- \| --- \| --- \| --- \| --- \| --- \| --- \| --- \| --- \| --- \| --- \| --- \| --- \| --- \| --- \| --- \| --- \| --- \| --- \| --- \| --- \| --- \| --- \| --- \| --- \| --- \| --- \| --- \| --- \| --- \| --- \| --- \| --- \| --- \| --- \| --- \| --- \| --- \| --- \| --- \| --- \| --- \| --- \| --- \| --- \| --- \| --- \| --- \| --- \| --- \| --- \| --- \| --- \| --- \| --- \| --- \| --- \| --- \| --- \| --- \| --- \| --- \| --- \| --- \| --- \| --- \| --- \| --- \| --- \| --- \| --- \| --- \| --- \| --- \| --- \| --- \| --- \| --- \| --- \| --- \| --- \| --- \| --- \| --- \| --- \| --- \| --- \| --- \| --- \| --- \| --- \| --- \| --- \| --- \| --- \| --- \| --- \| --- \| --- \| --- \| --- \| --- \| --- \| --- \| --- \| --- \| --- \| --- \| --- \| --- \| --- \| --- \| --- \| --- \| --- \| --- \| --- \| --- \| --- \| --- \| --- \| --- \| --- \| --- \| --- \| --- \| --- \| --- \| --- \| --- \| --- \| --- \| --- \| --- \| --- \| --- \| --- \| --- \| --- \| --- \| --- \| --- \| --- \| --- \| --- \| --- \| --- \| --- \| --- \| --- \| --- \| --- \| --- \| --- \| --- \| --- \| --- \| --- \| --- \| --- \| --- \| --- \| --- \| --- \| --- \| --- \| --- \| --- \| --- \| --- \| --- \| --- \| --- \| --- \| --- \| --- \| --- \| --- \| --- \| --- \| --- \| --- \| --- \| --- \| --- \| --- \| --- \| --- \| --- \| --- \| --- \| --- \| --- \| --- \| --- \| --- \| --- \| --- \| --- \| --- \| --- \| --- \| --- \| --- \| --- \| --- \| --- \| --- \| --- \| --- \| --- \| --- \| --- \| --- \| --- \| --- \| --- \| --- \| --- \| --- \| --- \| --- \| --- \| --- \| --- \| --- \| --- \| --- \| --- \| --- \| --- \| --- \| --- \| --- \| --- \| --- \| --- \| --- \| --- \| --- \| --- \| --- \| --- \| --- \| --- \| --- \| --- \| --- \| --- \| --- \| --- \| --- \| --- \| --- \| --- \| --- \| --- \| --- \| --- \| --- \| --- \| --- \| --- \| --- \| --- \| --- \| --- \| --- \| --- \| --- \| --- \| --- \| --- \| --- \| --- \| --- \| --- \| --- \| --- \| --- \| --- \| --- \| --- \| --- \| --- \| --- \| --- \| --- \| --- \| --- \| --- \| --- \| --- \| --- \| --- \| --- \| --- \| --- \| --- \| --- \| --- \| --- \| --- \| --- \| --- \| --- \| --- \| --- \| --- \| --- \| --- \| --- \| --- \| --- \| --- \| --- \| --- \| --- \| --- \| --- \| --- \| --- \| --- \| --- \| --- \| --- \| --- \| --- \| --- \| --- \| |
| --- | --- | --- | --- | --- | --- | --- | --- | --- | --- | --- | --- | --- | --- | --- | --- | --- | --- | --- | --- | --- | --- | --- | --- | --- | --- | --- | --- | --- | --- | --- | --- | --- | --- | --- | --- | --- | --- | --- | --- | --- | --- | --- | --- | --- | --- | --- | --- | --- | --- | --- | --- | --- | --- | --- | --- | --- | --- | --- | --- | --- | --- | --- | --- | --- | --- | --- | --- | --- | --- | --- | --- | --- | --- | --- | --- | --- | --- | --- | --- | --- | --- | --- | --- | --- | --- | --- | --- | --- | --- | --- | --- | --- | --- | --- | --- | --- | --- | --- | --- | --- | --- | --- | --- | --- | --- | --- | --- | --- | --- | --- | --- | --- | --- | --- | --- | --- | --- | --- | --- | --- | --- | --- | --- | --- | --- | --- | --- | --- | --- | --- | --- | --- | --- | --- | --- | --- | --- | --- | --- | --- | --- | --- | --- | --- | --- | --- | --- | --- | --- | --- | --- | --- | --- | --- | --- | --- | --- | --- | --- | --- | --- | --- | --- | --- | --- | --- | --- | --- | --- | --- | --- | --- | --- | --- | --- | --- | --- | --- | --- | --- | --- | --- | --- | --- | --- | --- | --- | --- | --- | --- | --- | --- | --- | --- | --- | --- | --- | --- | --- | --- | --- | --- | --- | --- | --- | --- | --- | --- | --- | --- | --- | --- | --- | --- | --- | --- | --- | --- | --- | --- | --- | --- | --- | --- | --- | --- | --- | --- | --- | --- | --- | --- | --- | --- | --- | --- | --- | --- | --- | --- | --- | --- | --- | --- | --- | --- | --- | --- | --- | --- | --- | --- | --- | --- | --- | --- | --- | --- | --- | --- | --- | --- | --- | --- | --- | --- | --- | --- | --- | --- | --- | --- | --- | --- | --- | --- | --- | --- | --- | --- | --- | --- | --- | --- | --- | --- | --- | --- | --- | --- | --- | --- | --- | --- | --- | --- | --- | --- | --- | --- | --- | --- | --- | --- | --- | --- | --- | --- | --- | --- | --- | --- | --- | --- | --- | --- | --- | --- | --- | --- | --- | --- | --- | --- | --- | --- | --- | --- | --- | --- | --- | --- | --- | --- | --- | --- | --- | --- | --- | --- | --- | --- | --- | --- | --- | --- | --- | --- | --- | --- | --- | --- | --- | --- | --- | --- | --- | --- | --- | --- | --- | --- | --- | --- | --- | --- | --- | --- | --- | --- | --- | --- | --- | --- | --- | --- | --- | --- | --- | --- | --- | --- | --- | --- | --- | --- | --- | --- | --- | --- | --- | --- | --- | --- | --- | --- | --- | --- | --- | --- | --- | --- | --- | --- | --- | --- | --- | --- | --- | --- | --- | --- | --- | --- | --- | --- | --- | --- | --- | --- | --- | --- | --- | --- | --- | --- | --- | --- | --- | --- | --- | --- | --- | --- | --- | --- | --- | --- | --- | --- | --- | --- | --- | --- | --- | --- | --- | --- | --- | --- | --- | --- | --- | --- | --- | --- | --- | --- | --- | --- | --- | --- | --- | --- | --- | --- | --- | --- | --- | --- | --- | --- | --- | --- | --- | --- | --- | --- | --- | --- | --- | --- | --- | --- | --- | --- | --- | --- | --- | --- | --- | --- | --- | --- | --- | --- | --- | --- | --- | --- | --- | --- | --- | --- | --- | --- | --- | --- | --- | --- | --- | --- | --- | --- | --- | --- | --- | --- | --- | --- | --- | --- | --- | --- | --- | --- | --- | --- | --- | --- | --- | --- | --- | --- | --- | --- | --- | --- | --- | --- | --- | --- | --- | --- | --- | --- | --- | --- | --- | --- | --- | --- | --- | --- | --- | --- | --- | --- | --- | --- | --- | --- | --- | --- | --- | --- | --- | --- | --- | --- | --- | --- | --- | --- | --- | --- | --- | --- | --- | --- | --- | --- | --- | --- | --- | --- | --- | --- | --- | --- | --- | --- | --- | --- | --- | --- | --- | --- | --- | --- | --- | --- | --- | --- | --- | --- | --- | --- | --- | --- | --- | --- | --- | --- | --- | --- | --- | --- | --- | --- | --- | --- | --- | --- | --- | --- | --- | --- | --- | --- | --- | --- | --- | --- | --- | --- | --- | --- | --- | --- | --- | --- | --- | --- | --- | --- | --- | --- | --- | --- | --- | --- | --- | --- | --- | --- | --- | --- | --- | --- | --- | --- | --- | --- | --- | --- | --- | --- | --- | --- | --- | --- | --- | --- | --- | --- | --- | --- | --- | --- | --- | --- | --- | --- | --- | --- | --- | --- | --- | --- | --- | --- | --- | --- | --- | --- | --- | --- | --- | --- | --- | --- | --- | --- | --- | --- | --- | --- | --- | --- | --- | --- | --- | --- | --- | --- | --- | --- | --- | --- | --- | --- | --- | --- | --- | --- | --- | --- | --- | --- | --- | --- | --- | --- | --- | --- | --- | --- | --- | --- | --- | --- | --- | --- | --- | --- | --- | --- | --- | --- | --- | --- | --- | --- | --- | --- | --- | --- | --- | --- | --- | --- | --- | --- | --- | --- | --- | --- | --- | --- | --- | --- | --- | --- | --- | --- | --- | --- | --- | --- | --- | --- | --- | --- | --- | --- | --- | --- | --- | --- | --- | --- | --- | --- | --- | --- | --- | --- | --- | --- | --- | --- | --- | --- | --- | --- | --- | --- | --- | --- | --- | --- | --- | --- | --- | --- | --- | --- | --- | --- | --- | --- | --- | --- | --- | --- | --- | --- | --- | --- | --- | --- | --- | --- | --- | --- | --- | --- | --- | --- | --- | --- | --- | --- | --- | --- | --- | --- | --- | --- | --- | --- | --- | --- | --- | --- | --- | --- | --- | --- | --- | --- | --- | --- | --- | --- | --- | --- | --- | --- | --- | --- | --- | --- | --- | --- | --- | --- | --- | --- | --- | --- | --- | --- | --- | --- | --- | --- | --- | --- | --- | --- | --- | --- | --- | --- | --- | --- | --- | --- | --- | --- | --- | --- | --- | --- | --- | --- | --- | --- | --- | --- | --- | --- | --- | --- | --- | --- | --- | --- | --- | --- | --- | --- | --- | --- | --- | --- | --- | --- | --- | --- | --- | --- | --- | --- | --- | --- | --- | --- | --- | --- | --- | --- | --- | --- | --- | --- | --- | --- | --- | --- | --- | --- | --- | --- | --- | --- | --- | --- | --- | --- | --- | --- | --- | --- | --- | --- | --- | --- | --- | --- | --- | --- | --- | --- | --- | --- | --- | --- | --- | --- | --- | --- | --- | --- | --- | --- | --- | --- | --- | --- | --- | --- | --- | --- | --- | --- | --- | --- | --- | --- | --- | --- | --- | --- | --- | --- | --- | --- | --- | --- | --- | --- | --- | --- | --- | --- | --- | --- | --- | --- | --- | --- | --- | --- | --- | --- | --- | --- | --- | --- | --- | --- | --- | --- | --- | --- | --- | --- | --- | --- | --- | --- | --- | --- | --- | --- | --- | --- | --- | --- | --- | --- | --- | --- | --- | --- | --- | --- | --- | --- | --- | --- | --- | --- | --- | --- | --- | --- | --- | --- | --- | --- | --- | --- | --- | --- | --- | --- | --- | --- | --- | --- | --- | --- | --- | --- | --- | --- | --- | --- | --- | --- | --- | --- | --- | --- | --- | --- | --- | --- | --- | --- | --- | --- | --- | --- | --- | --- | --- | --- | --- | --- | --- | --- | --- | --- | --- | --- | --- | --- | --- | --- | --- | --- | --- | --- | --- | --- | --- | --- | --- | --- | --- | --- | --- | --- | --- | --- | --- | --- | --- | --- | --- | --- | --- | --- | --- | --- | --- | --- | --- | --- | --- | --- | --- | --- | --- | --- | --- | --- | --- | --- | --- | --- | --- | --- | --- | --- | --- | --- | --- | --- | --- | --- | --- | --- | --- | --- | --- | --- | --- | --- | --- | --- | --- | --- | --- | --- | --- | --- | --- | --- | --- | --- | --- | --- | --- | --- | --- | --- | --- | --- | --- | --- | --- | --- | --- | --- | --- | --- | --- | --- | --- | --- | --- | --- | --- | --- | --- | --- | --- | --- | --- | --- | --- | --- | --- | --- | --- | --- | --- | --- | --- | --- | --- | --- | --- | --- | --- | --- | --- | --- | --- | --- | --- | --- | --- | --- | --- | --- | --- | --- | --- | --- | --- | --- | --- | --- | --- | --- | --- | --- | --- | --- | --- | --- | --- | --- | --- | --- | --- | --- | --- | --- | --- | --- | --- | --- | --- | --- | --- | --- | --- | --- | --- | --- | --- | --- | --- | --- | --- | --- | --- | --- | --- | --- | --- | --- | --- | --- | --- | --- | --- | --- | --- | --- | --- | --- | --- | --- | --- | --- | --- | --- | --- | --- | --- | --- | --- | --- | --- | --- | --- | --- | --- | --- | --- | --- | --- | --- | --- | --- | --- | --- | --- | --- | --- | --- | --- | --- | --- | --- | --- | --- | --- | --- | --- | --- | --- | --- | --- | --- | --- | --- | --- | --- | --- | --- | --- | --- | --- | --- | --- | --- | --- | --- | --- | --- | --- | --- | --- | --- | --- | --- | --- | --- | --- | --- | --- | --- | --- | --- | --- | --- | --- | --- | --- | --- | --- | --- | --- | --- | --- | --- | --- | --- | --- | --- | --- | --- | --- | --- | --- | --- | --- | --- | --- | --- | --- | --- | --- | --- | --- | --- | --- | --- | --- | --- | --- | --- | --- | --- | --- | --- | --- | --- | --- | --- | --- | --- | --- | --- | --- | --- | --- | --- | --- | --- | --- | --- | --- | --- | --- | --- | --- | --- | --- | --- | --- | --- | --- | --- | --- | --- | --- | --- | --- | --- | --- | --- | --- | --- | --- | --- | --- | --- | --- | --- | --- | --- | --- | --- | --- | --- | --- | --- | --- | --- | --- | --- | --- | --- | --- | --- | --- | --- | --- | --- | --- | --- | --- | --- | --- | --- | --- | --- | --- | --- | --- | --- | --- | --- | --- | --- | --- | --- | --- | --- | --- | --- | --- | --- | --- | --- | --- | --- | --- | --- | --- | --- | --- | --- | --- | --- | --- | --- | --- | --- | --- | --- | --- | --- | --- | --- | --- | --- | --- | --- | --- | --- | --- | --- | --- | --- | --- | --- | --- | --- | --- | --- | --- | --- | --- | --- | --- | --- | --- | --- | --- | --- | --- | --- | --- | --- | --- | --- | --- | --- | --- | --- | --- | --- | --- | --- | --- | --- | --- | --- | --- | --- | --- | --- | --- | --- | --- | --- | --- | --- | --- | --- | --- | --- | --- | --- | --- | --- | --- | --- | --- | --- | --- | --- | --- | --- | --- | --- | --- | --- | --- | --- | --- | --- | --- | --- | --- | --- | --- | --- | --- | --- | --- | --- | --- | --- | --- | --- | --- | --- | --- | --- | --- | --- | --- | --- | --- | --- | --- | --- | --- | --- | --- | --- | --- | --- | --- | --- | --- | --- | --- | --- | --- | --- | --- | --- | --- | --- | --- | --- | --- | --- | --- | --- | --- | --- | --- | --- | --- | --- | --- | --- | --- | --- | --- | --- | --- | --- | --- | --- | --- | --- | --- | --- | --- | --- | --- | --- | --- | --- | --- | --- | --- | --- | --- | --- | --- | --- | --- | --- | --- | --- | --- | --- | --- | --- | --- | --- | --- | --- | --- | --- | --- | --- | --- | --- | --- | --- | --- | --- | --- | --- | --- | --- | --- | --- | --- | --- | --- | --- | --- | --- | --- | --- | --- | --- | --- | --- | --- | --- | --- | --- | --- | --- | --- | --- | --- | --- | --- | --- | --- | --- | --- | --- | --- | --- | --- | --- | --- | --- | --- | --- | --- | --- | --- | --- | --- | --- | --- | --- | --- | --- | --- | --- | --- | --- | --- | --- | --- | --- | --- | --- | --- | --- | --- | --- | --- | --- | --- | --- | --- | --- | --- | --- | --- | --- | --- | --- | --- | --- | --- | --- | --- | --- | --- | --- | --- | --- | --- | --- | --- | --- | --- | --- | --- | --- | --- | --- | --- | --- | --- | --- | --- | --- | --- | --- | --- | --- | --- | --- | --- | --- | --- | --- | --- | --- | --- | --- | --- | --- | --- | --- | --- | --- | --- | --- | --- | --- | --- | --- | --- | --- | --- | --- | --- | --- | --- | --- | --- | --- | --- | --- | --- | --- | --- | --- | --- | --- | --- | --- | --- | --- | --- | --- | --- | --- | --- | --- | --- | --- | --- | --- | --- | --- | --- | --- | --- | --- | --- | --- | --- | --- | --- | --- | --- | --- | --- | --- | --- | --- | --- | --- | --- | --- | --- | --- | --- | --- | --- | --- | --- | --- | --- | --- | --- | --- | --- | --- | --- | --- | --- | --- | --- | --- | --- | --- | --- | --- | --- | --- | --- | --- | --- | --- | --- | --- | --- | --- | --- | --- | --- | --- | --- | --- | --- | --- | --- | --- | --- | --- | --- | --- | --- | --- | --- | --- | --- | --- | --- | --- | --- | --- | --- | --- | --- | --- | --- | --- | --- | --- | --- | --- | --- | --- | --- | --- | --- | --- | --- | --- | --- | --- | --- | --- | --- | --- | --- | --- | --- | --- | --- | --- | --- | --- | --- | --- | --- | --- | --- | --- | --- | --- | --- | --- | --- | --- | --- | --- | --- | --- | --- | --- | --- | --- | --- | --- | --- | --- | --- | --- | --- | --- | --- | --- | --- | --- | --- | --- | --- | --- | --- | --- | --- | --- | --- | --- | --- | --- | --- | --- | --- | --- | --- | --- | --- | --- | --- | --- | --- | --- | --- | --- | --- | --- | --- | --- | --- | --- | --- | --- | --- | --- | --- | --- | --- | --- | --- | --- | --- | --- | --- | --- | --- | --- | --- | --- | --- | --- | --- | --- | --- | --- | --- | --- | --- | --- | --- | --- | --- | --- | --- | --- | --- | --- | --- | --- | --- | --- | --- | --- | --- | --- | --- | --- | --- | --- | --- | --- | --- | --- | --- | --- | --- | --- | --- | --- | --- | --- | --- | --- | --- | --- | --- | --- | --- | --- | --- | --- | --- | --- | --- | --- | --- | --- | --- | --- | --- | --- | --- | --- | --- | --- | --- | --- | --- | --- | --- | --- | --- | --- | --- | --- | --- | --- | --- | --- | --- | --- | --- | --- | --- | --- | --- | --- | --- | --- | --- | --- | --- | --- | --- | --- | --- | --- | --- | --- | --- | --- | --- | --- | --- | --- | --- | --- | --- | --- | --- | --- | --- | --- | --- | --- | --- | --- | --- | --- | --- | --- | --- | --- | --- | --- | --- | --- | --- | --- | --- | --- | --- | --- | --- | --- | --- | --- | --- | --- | --- | --- | --- | --- | --- | --- | --- | --- | --- | --- | --- | --- | --- | --- | --- | --- | --- | --- | --- | --- | --- | --- | --- | --- | --- | --- | --- | --- | --- | --- | --- | --- | --- | --- | --- | --- | --- | --- | --- | --- | --- | --- | --- | --- | --- | --- | --- | --- | --- | --- | --- | --- | --- | --- | --- | --- | --- | --- | --- | --- | --- | --- | --- | --- | --- | --- | --- | --- | --- | --- | --- | --- | --- | --- | --- | --- | --- | --- | --- | --- | --- | --- | --- | --- | --- | --- | --- | --- | --- | --- | --- | --- | --- | --- | --- | --- | --- | --- | --- | --- | --- | --- | --- | --- | --- | --- | --- | --- | --- | --- | --- | --- | --- | --- | --- | --- | --- | --- | --- | --- | --- | --- | --- | --- | --- | --- | --- | --- | --- | --- | --- | --- | --- | --- | --- | --- | --- | --- | --- | --- | --- | --- | --- | --- | --- | --- | --- | --- | --- | --- | --- | --- | --- | --- | --- | --- | --- | --- | --- | --- | --- | --- | --- | --- | --- | --- | --- | --- | --- | --- | --- | --- | --- | --- | --- | --- | --- | --- | --- | --- | --- | --- | --- | --- | --- | --- | --- | --- | --- | --- | --- | --- | --- | --- | --- | --- | --- | --- | --- | --- | --- | --- | --- | --- | --- | --- | --- | --- | --- | --- | --- | --- | --- | --- | --- | --- | --- | --- | --- | --- | --- | --- | --- | --- | --- | --- | --- | --- | --- | --- | --- | --- | --- | --- | --- | --- | --- | --- | --- | --- | --- | --- | --- | --- | --- | --- | --- | --- | --- | --- | --- | --- | --- | --- | --- | --- | --- | --- | --- | --- | --- | --- | --- | --- | --- | --- | --- | --- | --- | --- | --- | --- | --- | --- | --- | --- | --- | --- | --- | --- | --- | --- | --- | --- | --- | --- | --- | --- | --- | --- | --- | --- | --- | --- | --- | --- | --- | --- | --- | --- | --- | --- | --- | --- | --- | --- | --- | --- | --- | --- | --- | --- | --- | --- | --- | --- | --- | --- | --- | --- | --- | --- | --- | --- | --- | --- | --- | --- | --- | --- | --- | --- | --- | --- | --- | --- | --- | --- | --- | --- | --- | --- | --- | --- | --- | --- | --- | --- | --- | --- | --- | --- | --- | --- | --- | --- | --- | --- | --- | --- | --- | --- | --- | --- | --- | --- | --- | --- | --- | --- | --- | --- | --- | --- | --- | --- | --- | --- | --- | --- | --- | --- | --- | --- | --- | --- | --- | --- | --- | --- | --- | --- | --- | --- | --- | --- | --- | --- | --- | --- | --- | --- | --- | --- | --- | --- | --- | --- | --- | --- | --- | --- | --- | --- | --- | --- | --- | --- | --- | --- | --- | --- | --- | --- | --- | --- | --- | --- | --- | --- | --- | --- | --- | --- | --- | --- | --- | --- | --- | --- | --- | --- | --- | --- | --- | --- | --- | --- | --- | --- | --- | --- | --- | --- | --- | --- | --- | --- | --- | --- | --- | --- | --- | --- | --- | --- | --- | --- | --- | --- | --- | --- | --- | --- | --- | --- | --- | --- | --- | --- | --- | --- | --- | --- | --- | --- | --- | --- | --- | --- | --- | --- | --- | --- | --- | --- | --- | --- | --- | --- | --- | --- | --- | --- | --- | --- | --- | --- | --- | --- | --- | --- | --- | --- | --- | --- | --- | --- | --- | --- | --- | --- | --- | --- | --- | --- | --- | --- | --- | --- | --- | --- | --- | --- | --- | --- | --- | --- | --- | --- | --- | --- | --- | --- | --- | --- | --- | --- | --- | --- | --- | --- | --- | --- | --- | --- | --- | --- | --- | --- | --- | --- | --- | --- | --- | --- | --- | --- | --- | --- | --- | --- | --- | --- | --- | --- | --- | --- | --- | --- | --- | --- | --- | --- | --- | --- | --- | --- | --- | --- | --- | --- | --- | --- | --- | --- | --- | --- | --- | --- | --- | --- | --- | --- | --- | --- | --- | --- | --- | --- | --- | --- | --- | --- | --- | --- | --- | --- | --- | --- | --- | --- | --- | --- | --- | --- | --- | --- | --- | --- | --- | --- | --- | --- | --- | --- | --- | --- | --- | --- | --- | --- | --- | --- | --- | --- | --- | --- | --- | --- | --- | --- | --- | --- | --- | --- | --- | --- | --- | --- | --- | --- | --- | --- | --- | --- | --- | --- | --- | --- | --- | --- | --- | --- | --- | --- | --- | --- | --- | --- | --- | --- | --- | --- | --- | --- | --- | --- | --- | --- | --- | --- | --- | --- | --- | --- | --- | --- | --- | --- | --- | --- | --- | --- | --- | --- | --- | --- | --- | --- | --- | --- | --- | --- | --- | --- | --- | --- | --- | --- | --- | --- | --- | --- | --- | --- | --- | --- | --- | --- | --- | --- | --- | --- | --- | --- | --- | --- | --- | --- | --- | --- | --- | --- | --- | --- | --- | --- | --- | --- | --- | --- | --- | --- | --- | --- | --- | --- | --- | --- | --- | --- | --- | --- | --- | --- | --- | --- | --- | --- | --- | --- | --- | --- | --- | --- | --- | --- | --- | --- | --- | --- | --- | --- | --- | --- | --- | --- | --- | --- | --- | --- | --- | --- | --- | --- | --- | --- | --- | --- | --- | --- | --- | --- | --- | --- | --- | --- | --- | --- | --- | --- | --- | --- | --- | --- | --- | --- | --- | --- | --- | --- | --- | --- | --- | --- | --- | --- | --- | --- | --- | --- | --- | --- | --- | --- | --- | --- | --- | --- | --- | --- | --- | --- | --- | --- | --- | --- | --- | --- | --- | --- | --- | --- | --- | --- | --- | --- | --- | --- | --- | --- | --- | --- | --- | --- | --- | --- | --- | --- | --- | --- | --- | --- | --- | --- | --- | --- | --- | --- | --- | --- | --- | --- | --- | --- | --- | --- | --- | --- | --- | --- | --- | --- | --- | --- | --- | --- | --- | --- | --- | --- | --- | --- | --- | --- | --- | --- | --- | --- | --- | --- | --- | --- | --- | --- | --- | --- | --- | --- | --- | --- | --- | --- | --- | --- | --- | --- | --- | --- | --- | --- | --- | --- | --- | --- | --- | --- | --- | --- | --- | --- | --- | --- | --- | --- | --- | --- | --- | --- | --- | --- | --- | --- | --- | --- | --- | --- | --- | --- | --- | --- | --- | --- | --- | --- | --- | --- | --- | --- | --- | --- | --- | --- | --- | --- | --- | --- | --- | --- | --- | --- | --- | --- | --- | --- | --- | --- | --- | --- | --- | --- | --- | --- | --- | --- | --- | --- | --- | --- | --- | --- | --- | --- | --- | --- | --- | --- | --- | --- | --- | --- | --- | --- | --- | --- | --- | --- | --- | --- | --- | --- | --- | --- | --- | --- | --- | --- | --- | --- | --- | --- | --- | --- | --- | --- | --- | --- | --- | --- | --- | --- | --- | --- | --- | --- | --- | --- | --- | --- | --- | --- | --- | --- | --- | --- | --- | --- | --- | --- | --- | --- | --- | --- | --- | --- | --- | --- | --- | --- | --- | --- | --- | --- | --- | --- | --- | --- | --- | --- | --- | --- | --- | --- | --- | --- | --- | --- | --- | --- | --- | --- | --- | --- | --- | --- | --- | --- | --- | --- | --- | --- | --- | --- | --- | --- | --- | --- | --- | --- | --- | --- | --- | --- | --- | --- | --- | --- | --- | --- | --- | --- | --- | --- | --- | --- | --- | --- | --- | --- | --- | --- | --- | --- | --- | --- | --- | --- | --- | --- | --- | --- | --- | --- | --- | --- | --- | --- | --- | --- | --- | --- | --- | --- | --- | --- | --- | --- | --- | --- | --- | --- | --- | --- | --- | --- | --- | --- | --- | --- | --- | --- | --- | --- | --- | --- | --- | --- | --- | --- | --- | --- | --- | --- | --- | --- | --- | --- | --- | --- | --- | --- | --- | --- | --- | --- | --- | --- | --- | --- | --- | --- | --- | --- | --- | --- | --- | --- | --- | --- | --- | --- | --- | --- | --- | --- | --- | --- | --- | --- | --- | --- | --- | --- | --- | --- | --- | --- | --- | --- | --- | --- | --- | --- | --- | --- | --- | --- | --- | --- | --- | --- | --- | --- | --- | --- | --- | --- | --- | --- | --- | --- | --- | --- | --- | --- | --- | --- | --- | --- | --- | --- | --- | --- | --- | --- | --- | --- | --- | --- | --- | --- | --- | --- | --- | --- | --- | --- | --- | --- | --- | --- | --- | --- | --- | --- | --- | --- | --- | --- | --- | --- | --- | --- | --- | --- | --- | --- | --- | --- | --- | --- | --- | --- | --- | --- | --- | --- | --- | --- | --- | --- | --- | --- | --- | --- | --- | --- | --- | --- | --- | --- | --- | --- | --- | --- | --- | --- | --- | --- | --- | --- | --- | --- | --- | --- | --- | --- | --- | --- | --- | --- | --- | --- | --- | --- | --- | --- | --- | --- | --- | --- | --- | --- | --- | --- | --- | --- | --- | --- | --- | --- | --- | --- | --- | --- | --- | --- | --- | --- | --- | --- | --- | --- | --- | --- | --- | --- | --- | --- | --- | --- | --- | --- | --- | --- | --- | --- | --- | --- | --- | --- | --- | --- | --- | --- | --- | --- | --- | --- | --- | --- | --- | --- | --- | --- | --- | --- | --- | --- | --- | --- | --- | --- | --- | --- | --- | --- | --- | --- | --- | --- | --- | --- | --- | --- | --- | --- | --- | --- | --- | --- | --- | --- | --- | --- | --- | --- | --- | --- | --- | --- | --- | --- | --- | --- | --- | --- | --- | --- | --- | --- | --- | --- | --- | --- | --- | --- | --- | --- | --- | --- | --- | --- |
